# Small-Angle X‑ray Scattering Monitoring of Porosity Evolution in Iron–Nitrogen–Carbon Electrocatalysts

**DOI:** 10.1021/acsnano.5c14955

**Published:** 2025-11-14

**Authors:** Rutha Jäger, Patrick Teppor, Armin Hoell, Uwe Keiderling, Christian Gollwitzer, Olga Volobujeva, Jaan Aruväli, Zdravko Kochovski, Eneli Härk

**Affiliations:** † Institute of Chemistry, Chair of Physical Chemistry, 37546 University of Tartu, Ravila 14A, 50411 Tartu, Estonia; ‡ Department Structure and Dynamics of Energy Materials, 28340 Helmholtz-Zentrum Berlin für Materialien und Energie, Hahn-Meitner-Platz 1, 14109 Berlin, Germany; § Department Experiment Control and Data Acquisition, Helmholtz-Zentrum Berlin für Materialien und Energie, Hahn-Meitner-Platz 1, 14109 Berlin, Germany; ∥ 54367 Physikalisch-Technische Bundesanstalt (PTB), Abbestr. 2-12, 10587 Berlin, Germany; ⊥ Department of Materials and Environmental Technology, 54561 Tallinn University of Technology, Ehitajate tee 5, 19086 Tallinn, Estonia; # Institute of Ecology and Earth Sciences, Department of Geology, University of Tartu, Ravila 14A, 50411 Tartu, Estonia; ∇ Institute Electrochemical Energy Storage, Helmholtz-Zentrum Berlin für Materialien und Energie, Hahn-Meitner-Platz 1, 14109 Berlin, Germany

**Keywords:** SAXS structural model-free analysis, Fe–N–C electrocatalysts, average graphene layer curvature, hierarchical porosity, hydroxyapatite

## Abstract

This study highlights the use of anomalous and small-angle X-ray scattering (ASAXS/SAXS) to monitor the evolution of micro- and mesoporosity during the synthesis of iron–nitrogen–carbon (Fe–N–C) electrocatalysts. A structural model-free SAXS approach enabled the determination of 13 structural parameters across five Fe–N–C electrocatalysts, compared to a commercial PMF-12704, Pajarito Powder. SAXS revealed hierarchical pore formation and structural changes spanning micro- to macroscales. Key features, such as pore curvature, porosity, and disorder, increased with synthesis modifications, correlating with enhanced oxygen reduction activity and reduced hydrogen peroxide yield. Notably, an average graphene layer curvature (*l*_R_–Ruland length) above 3 nm was critical for forming curved pore walls, which improves selectivity and favors the 4-electron oxygen reduction pathway to water. These findings highlight the pore curvature and hierarchical pore architecture as crucial design parameters, guiding the development of next-generation electrocatalysts with enhanced efficiency and selectivity for sustainable energy applications. Specifically, optimizing the mesopore size distribution, with an increased proportion of mesopores within the range from 6 to 27 nm, and improving the transition from micropores to mesopores and macropores are essential strategies.

## Graphical abstract

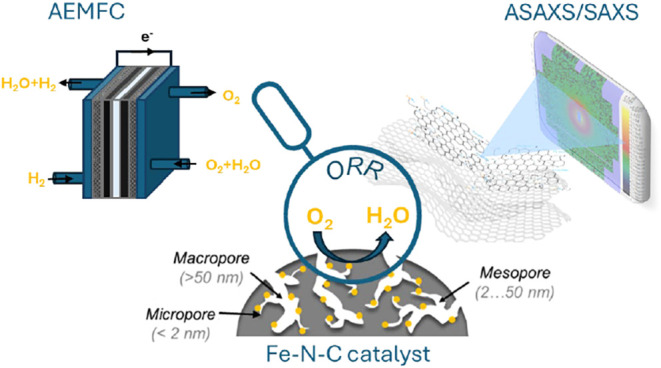

## Introduction

The widespread adoption of fuel cell technology in renewable energy systems is still hampered by its high cost. Costs are expected to decrease significantly as production capacity increases.^1,2^ Another critical challenge is the shortage of platinum, which is essential for fuel cell catalysts. If all mined platinum were used for fuel cells, supplies could be depleted by 2045.^3^ Consequently, extensive research is being conducted to develop alternative catalyst materials to ensure the viability of fuel cells on a larger scale. A promising alternative to platinum-based catalysts is a nonprecious metal catalyst consisting mainly of iron, nitrogen, and carbon, and its structure plays a crucial role in the activity and selectivity of the oxygen reduction reaction (ORR). Iron–nitrogen–carbon (Fe–N–C) electrocatalysts often struggle to achieve the 4-electron pathway for oxygen reduction to water. Hydrogen peroxide can degrade the catalyst or other fuel cell components, shortening the system’s life. Hossen et al.^4^ have noted that for anion-exchange membrane fuel cell (AEMFC) catalysts to be practical, the peroxide yield must remain below 5% at electrode potentials between 0.5 and 0.8 V. The optimized hierarchical porosity of the electrocatalyst with well-distributed active sites is needed to enhance the ORR selectivity and activity.^5^ Porosity serves two principal functions for nonprecious catalysts: ensuring efficient mass transport of reactants and products, similarly to Pt-based catalysts, and supporting active site formation. Hard templates, such as silica spheres, are utilized to create mesopores, thereby increasing the porosity of Fe–N–C electrocatalysts and providing optimal meso- and macroporosity for enhanced fuel cell performance.^6–10^ However, hazardous hydrogen fluoride is usually required to remove silica, prompting the search for safer alternatives. Therefore, a hydroxyapatite (HA) hard template, in addition to a pore modifier, ZnCl_2_, to obtain hierarchically porous (micro-, meso-, and macroporous) Fe–N–C catalyst materials is more ecologically meaningful and demonstrates excellent activity in alkaline solutions in three-electrode experiments and AEMFC tests.^11^

Several studies have demonstrated that the reactivity of Fe–N–C electrocatalysts is strongly influenced by the orientation of the local carbon structure, i.e., the carbon plane relative to the mesopores (2 nm < *d*_pore_ < 50 nm). Specifically, when the carbon basal plane is oriented toward the mesopores (“plane-exposed”), the formation of Fe–N_3_, Fe–N_4_, and Fe–N_4+1_ active sites is favored. In contrast, an “edge-exposed” configuration primarily leads to Fe–N_2_ sites and edge-located nitrogen moieties. Moreover, Fe–N_2+2_ configurations have been reported to act as bridges between two crystallites, with preferential micropore localization (*d*_pore_ < 2 nm).^5,12–14^ Thus, the optimal porosity of the electrocatalyst and the location of the active sites (bulk, edge, or between the sheets) are crucial. So far, it has been impossible to directly measure the active sites in real Fe–N–C electrocatalysts. This is mainly because the carbon layers are very thin and the number of active sites is relatively low, making it extremely hard for current characterization techniques to detect and identify specific types of sites. These challenges make it difficult to fully understand how the structure of these materials relates to their performance, indicating that better methods are still needed.^12–16^

Anomalous small-angle X-ray scattering (ASAXS) is an element-sensitive technique used to study changes in electron density within a sample. ASAXS and small-angle X-ray scattering (SAXS) are generally sensitive to average electron density fluctuations in size ranges from about 0.5 to several 100 nm, depending on instrumental conditions. Both methods are applied to various fields of material sciences, energy conversion, and storage sciences, and studies of electrocatalysts, such as precious metal catalysts in porous carbon matrix, including Pt, Au, Ir, Pd, and Ni.^17–25^ In electrocatalysts, it helps to identify nanosized structural differences and distinguish between the precious metal particles or pores and the carbon support. ASAXS is a reliable method for studying precious metal catalysts distributed on carbon supports, even when only small amounts of catalyst are present, down to a few tens of cubic millimeters at atomic fraction levels.^22^ This technique can also be used to look at specific parts of bimetallic catalysts (Pt­(Ni)/TiO_2_, Pt–Ru, Ru–Se, PtNi_6_, Ni–Cu), allowing the measurement of the size and surface area of each type of metal particle separately.^18,20,21,24,26^

Nondestructive ASAXS/SAXS, X-ray Absorption Near-Edge Structure (XANES), and X-ray Fluorescence (XRF) techniques enable fast measurements, allowing data to be collected from the same sample and at the same beamline, making it easier to understand the material without the need for separate experiments. While precious metals such as Pt, Pd, Ir, and Au are more commonly studied with ASAXS and SAXS due to their extensive catalytic applications, nonprecious metals such as Fe, Co, Ni, Cu, and Mn are increasingly being explored, especially for cost-effective and sustainable catalysis (e.g., in ORR, oxygen evolution reaction, hydrogen evolution reaction, and carbon dioxide reduction reaction).^27–31^ Until now, detailed ASAXS/SAXS studies have not been widely used for nonprecious metal catalysts. These materials are harder to study, because they can easily oxidize or form mixed phases. This is even more challenging when the metals are part of complex structures like nitrogen-doped carbon or electrocatalysts, such as Fe–N_3_, Fe–N_4_, and Fe–N_4+1_ active sites.^5,32^ The Fellinger group outlined the general principles for conventional metal nitrogen–carbon electrocatalyst synthesis, which typically involves heating metal, carbon, and nitrogen precursors in an inert atmosphere at temperatures ranging from approximately 750 to 1300 °C.^32^ Asset and Atanassov highlighted the distinction between in-plane and edge-located active sites.^5^ In this work, we propose that specific structural dimensions emerge as a result of modifying the synthesis conditions of the Fe–N/Cat-X electrocatalyst. Thus, we focus on the electrocatalyst structure, mainly increasing micro- and mesopores and adjusting the morphology (i.e., curvature), which improves selectivity and boosts the 4-electron ORR reaction pathway, i.e., creates the right structural environment for the conversion of H_2_O_2_ to H_2_O. Our structural model-free SAXS analysis provides in-depth insights into complex structure–electrochemical correlations. A structural model-free approach enables nonpredefined shape assumptions and requires only measured scattering data, making them particularly valuable for complex disordered structures, materials with irregular or heterogeneous pore morphologies, and systems where the actual structural geometry is unknown. This work aims to contribute further understanding of optimizing synthesis conditions to obtain active and selective nonprecious electrocatalysts for AEMFC.

## Results and Discussion

### Key Differences in Synthesis Conditions among Electrocatalysts

Hydroxyapatite (**HA**, Ca_10_(PO_4_)_6_(OH)_2_, ≥ 96%, (20 × 80 ± 10) nm particle size, Sigma-Aldrich) was introduced as a hard template for the synthesis of hierarchically porous Fe–N–C electrocatalysts, which showed excellent ORR activity under alkaline conditions onset potential: 0.96 V vs RHE (in Table 1) and achieved a peak power density of 1.06 W cm^–2^ in AEMFC tests.^11^ This study’s results place it among a selected group of catalysts, as reviewed by Hossen et al.,^4^ that exceeds the 1000 mW cm^–2^ threshold in AEMFC applications.

**Table 1:** An Overview of the Synthesis Conditions for the Fe–N/Cat-X Electrocatalysts

precursors	Fe–N/Cat-1	Fe–N/Cat-2	Fe–N/Cat-3	Fe–N/Cat-4	Fe–N/Cat-5
peat (g)	3	3	3	3	3
guanidine carbonate (g)	1	1	1	1	1
Fe(NO_3_)_3_·9H_2_O (g)	0.1	0.1	0.1	0.1	0.2
ZnCl_2_ (g)	2	4	4	4	4
hydroxyapatite (g)				4	4
pyrolysis temp (°C)	800	800	1000	800	1000

The sketched diamond-shaped comparative diagram enables the identification of key differences in preparation methods between these five separate catalysts and guides the reader through the article (Figure 1a). This diamond-shaped diagram does not represent a sequential synthesis where each catalyst builds upon the previous one. Instead, each **Fe–N/Cat-X** (1 through 5) has been synthesized independently, and the synthesis procedure is described in detail in our earlier work.^11^ Briefly, regarding the synthesis, the peat was first mixed with water and then dried and powdered. In a typical synthesis (**Fe–N/Cat-1**), all precursors (Table 1 and Supporting Information (SI)) were blended in 15 mL of isopropyl alcohol (IPA). The mixture underwent grinding cycles, high-shear mixing, and ultrasonic treatment. After drying and initial pyrolysis at 800 °C for 1 h under Ar flow, the powder was acid-washed with 1 M HNO_3_ for 8 h at 80 °C. A second pyrolysis step was followed at the same temperature, and the resulting catalyst powder was further processed in a mortar for 30 min.

**Figure 1:**
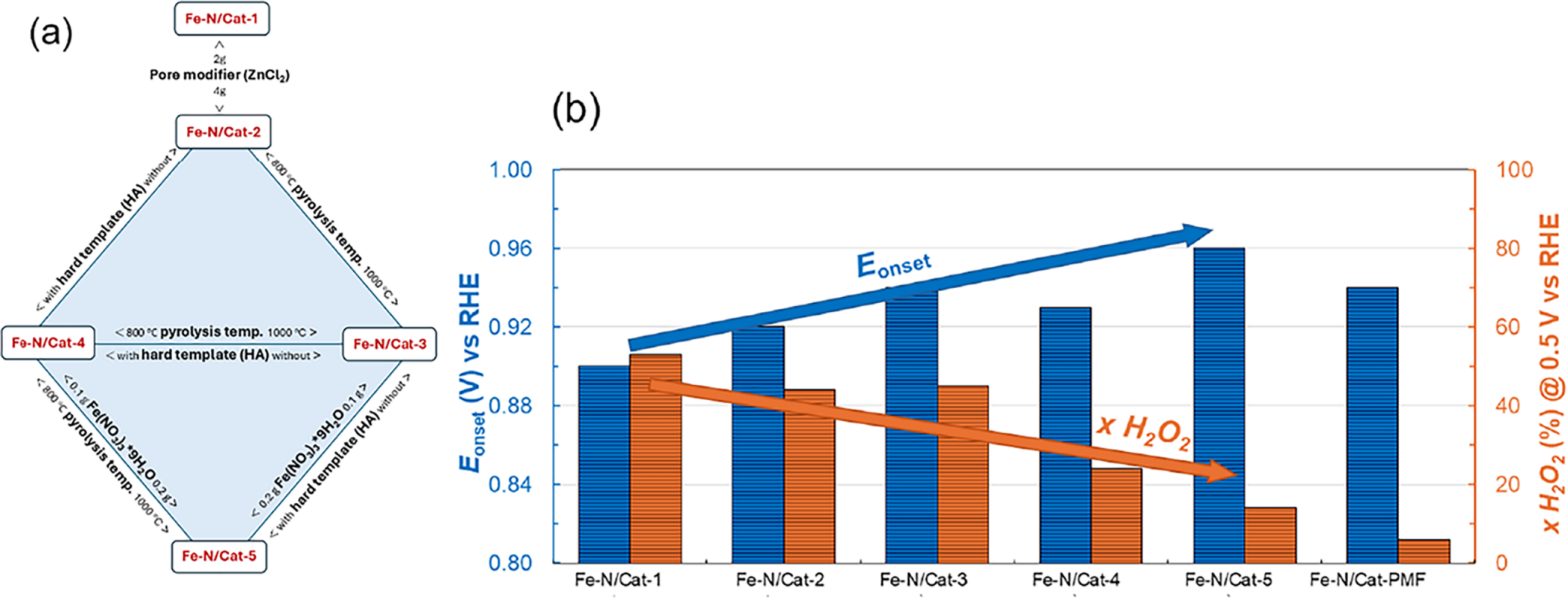
Synthesis conditions and electrochemical characterization. (a) The diamond-shaped diagram illustrates different Fe–N/Cat-X electrocatalysts and their synthesis conditions. (b) The onset potential values for the oxygen reduction reaction (ORR) relative to the reversible hydrogen electrode (RHE) and the peroxide yield at electrode potential 0.5 V vs RHE were evaluated for Fe–N/Cat-X catalysts. These values were compared with those of the commercial Fe–N/Cat-PMF reference catalyst using rotating ring-disk electrode (RRDE) experiments. The measurements were conducted in an O_2_-saturated 0.1 M KOH solution, with a potential scan range from 1.2 to 0.3 V vs RHE at a scan rate of 10 mV s^–1^ while maintaining a constant ring potential of 1.2 V vs RHE. Detailed information can be found in ref 11. The SI section briefly describes the RRDE measurement methodology, accompanied by Figure S1.

The links between the electrocatalysts (Figure 1a) indicate the key differences in the synthesis conditions between each **Fe–N/Cat-X**. In short, **Fe–N/Cat-1** and **Fe–N/Cat-2** differ in that **Fe–N/Cat-2** uses double the amount of ZnCl_2_ as a pore modifier in its synthesis. **Fe–N/Cat-2** and **Fe–N/Cat-3** differ in their pyrolysis temperature, with **Fe–N/Cat-3** being prepared at 1000 °C. Then, **Fe–N/Cat-4** differs from **Fe–N/Cat-2** in that a hard template (HA) is additionally used to synthesize **Fe–N/Cat-4**. Finally, the **Fe–N/Cat-5** catalyst can be compared with **Fe–N/Cat-4**; the difference between them is the amount of Fe salt in the synthesis process and the difference in pyrolysis temperature, while with **Fe–N/Cat-3**, in addition to the difference in Fe salt, there is also an addition of HA to the synthesis precursors, as shown in Figure 1a. A commercial Fe–N–C catalyst material (PMF-12704, Pajarito Powder, LLC) designated as **Fe–N/Cat-PMF** was used for comparison. According to electrochemical analysis, it was determined that among the materials in the diamond-shaped diagram, **Fe–N/Cat-5** was the most ORR active (highest onset potential) and selective (lowest peroxide yield) (Figure 1b). It was concluded that specific synthesis conditions improved the hierarchical porosity and enhanced the catalytic activity and selectivity. We answer the question of what structural peculiarities should be followed to achieve high activity and selectivity in the electrocatalyst.

Table 1 presents the refined synthesis conditions, and Table 2 presents the parameters obtained from various physical and electrochemical characterization methods, including the scanning electron microscope with energy-dispersive X-ray spectroscopy (SEM-EDS) results, which serve as an input for calculating the scattering contrast of each electrocatalyst using the Scattering Length Density Calculator, developed by the NIST Center for Neutron Research,^33^ essential for analyzing the SAXS data.

**Table 2:** Core Physical and Electrochemical Characterization Results for the Fe–N/Cat-X Electrocatalysts^a^

parameter	Fe–N/Cat-1	Fe–N/Cat-2	Fe–N/Cat-3	Fe–N/Cat-4	Fe–N/Cat-5	Fe–N/Cat-PMF
low-temperature N_2_ sorption analysis						
*S*_DFT_ (m^2^/g)	950	1220	1040	1150	1280	880
*V*_micro_/*V*_DFT_	100	88	93	64	73	20
scanning electron microscopy with energy-dispersive X-ray spectroscopy						
C (wt%)	73	80	83	66	81	83
O (wt%)	11	7.4	11	15	13	9.5
N (wt%)	8.7	8.8	3.9	6.5	3.1	6.6
Fe (wt%)	0.98	0.75	0.86	2.70	1.29	0.28
Zn (wt%)	0.51	0.78		2.17		
Ca (wt%)				4.89		
P (wt%)				2.64	1.73	
other residuals	W	Si	W, Si, S	Si, Al, Cl	Al, Mg	Cu
electrochemical characterization						
*E*_onset_ (V vs RHE)	0.90	0.92	0.94	0.93	0.96	0.94
*x* H_2_O_2_ yield (%)	53	44	45	25	14	6
*n*	2.9	3.1	3.1	3.5	3.8	3.9

a S_DFT_, specific surface area according to the SAIEUS pore size distribution from N_2_ sorption analysis; *V*_micro_/*V*_DFT_ describes the proportion of the volume of micropores (pores with a width of up to 2 nm) to the total volume of pores with a width of up to 30 nm according to N_2_ sorption analysis, see also Figure S2. The elemental compositions of the Fe–N/Cat-X catalysts were obtained using SEM-EDS; *E*_onset_, onset potential defined as the potential value at a current density of −0.1 mA cm^–2^ in ORR curves; *x* H_2_O_2_ yield at 0.5 V vs RHE—hydrogen peroxide yield obtained from RRDE measurement data (Figure S1); and *n* is the number of electrons transferred per molecule of oxygen obtained from RRDE data at 0.5 V vs RHE.

### Elemental Composition and Electronic Structural Properties of Electrocatalysts

Figure 2a shows the X-ray fluorescence spectra (XRF) which were obtained for **Fe–N/Cat-4**, **Fe–N/Cat-5**, and a commercial **Fe–N/Cat-PMF** electrocatalyst. The spectra were normalized to the intensity of the elastic peak at 10 keV. In addition to the electrocatalysts, the spectrum obtained on the sticky tape used for enclosing the sample is shown as the gray-shaded area. While reference-free XRF allows, in principle, the quantification of the elements in the sample,^34^ here, we show the uncalibrated spectra, which allow qualitative analysis only. As expected, the Fe Kα and Kβ fluorescence lines are the most prominent spectral features. In addition, the **Fe–N/Cat-4** electrocatalyst shows significant signals from the presence of Zn, Ca, and Cl. Of these, only Ca is also present in **Fe–N/Cat-5**, though at lower concentrations and along with a trace amount of Zn. The **Fe–N/Cat-PMF** electrocatalyst exhibits detectable signals from Ti and K attributed to the background and originating from the adhesive tape used during sample preparation, with no apparent contribution from the samples themselves.

**Figure 2:**
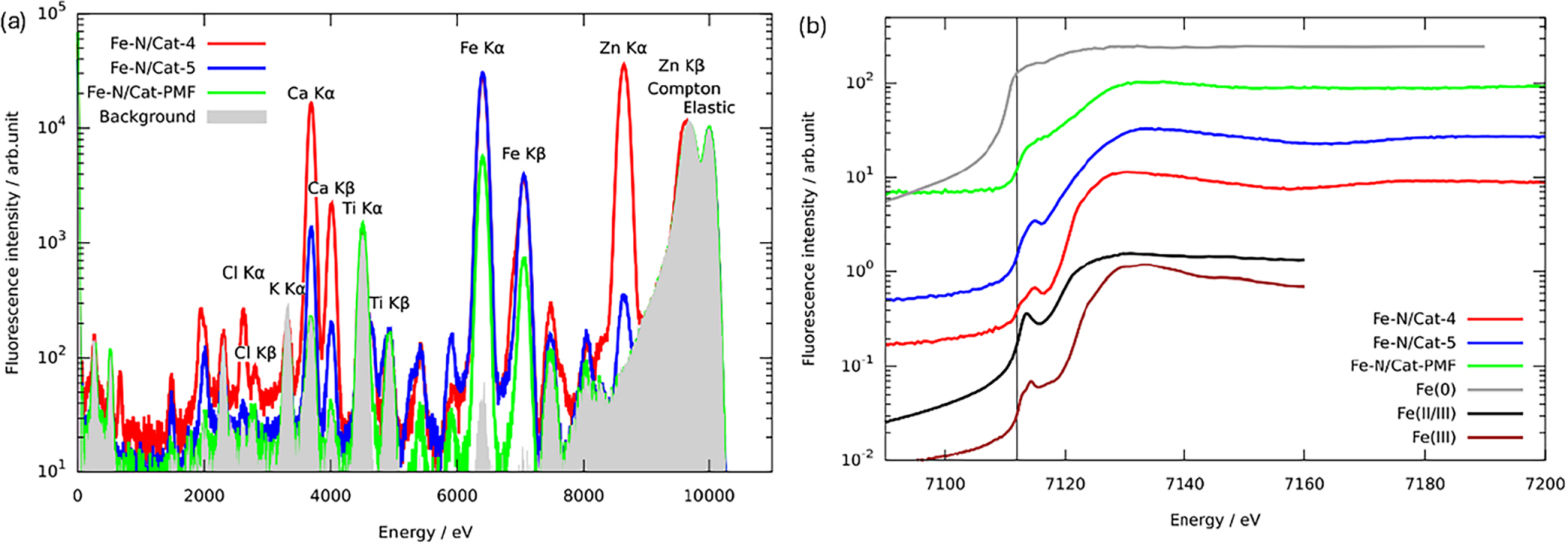
Electrocatalysts Fe–N/Cat-4, Fe–N/Cat-5, and Fe–N/Cat-PMF elemental composition and electronic structural properties. (a) X-ray fluorescence spectra. (b) X-ray absorption near-edge structure spectra. The vertical black line at 7112 eV marks the theoretical absorption edge position, corresponding to the Fe K-edge.

Additionally, X-ray absorption near-edge structure spectra (XANES) were collected across the Fe K-edge from 7090 to 7200 eV in steps of 0.5 eV with an exposure time of 10 s (Figure 2b). The intensity of the Fe Kα fluorescence was used to derive the XANES signal. Figure 2b shows results and recorded data on a 99.99% pure Fe metal foil^35^ (Goodfellow Cambridge Limited), Fe_3_O_4_ (magnetite powder, <5 μm, 95%, Merck), and γ-Fe_2_O_3_ (maghemite powder, <5 μm, ≥96%, Merck) powders for reference. As expected, all samples display a pre-edge peak at around 7112 eV, except for the pure Fe metal foil. This indicates that the iron in the samples is in an oxidized state. When the shape of the pre-edge peak is compared with the references, the peak of **Fe–N/Cat-4** is very similar to that of Fe_2_O_3_, indicating fully oxidized iron. The peak is more rounded for **Fe–N/Cat-5**, especially **Fe–N/Cat-PMF**, indicating a lower oxidation state. In the case of **Fe–N/Cat-5**, we can confidently state that our objective has been achieved, as the electrocatalyst contains selective and active centers. However, for **Fe–N/Cat-4**, as confirmed by XANES, we observe the presence of mixed phases and oxidized iron forms (Fe_2_O_3_), which reduce the electrocatalyst’s selectivity, indicating that the iron is fully oxidized.

### Anomalous Small-Angle X-ray Scattering and Structural Model-Free Analysis

Characterization methods, including transmission electron microscopy (Figure S3), X-ray photoelectron spectroscopy (Table S1), SEM-EDS, and X-ray diffraction method (Figure S4), have confirmed the presence of iron embedded in a carbon matrix. The objective was to locate and characterize the iron particles separately from the carbon structure using ASAXS. This approach is necessary because conventional SAXS primarily detects overall electron density contrast between components, making it challenging to distinguish between iron particles caused by scattering, pores, or possible third kinds of nanostructures. In a typical ASAXS measurement, different scattering patterns are recorded at different X-ray energies near the absorption edge of the target element, Fe, where iron’s effective electron density changes. This method has been successfully applied to study precious metal catalysts in porous carbon matrixes, including Pt, Pt–Ru, Ru–Se, Au, Ir, Pd, and Ni, where the particles are in the metallic state and at least nanometer-sized. However, to our knowledge, nonprecious metal catalysts have not yet been studied using the SAXS structural model-free analysis or ASAXS. The ASAXS measurement results are in Figure 3a,b for the best-performing ORR catalysts, **Fe–N/Cat-5** and **Fe–N/Cat-PMF** samples. The small-angle scattering intensities are measured with different X-ray energies ranging from 6692.0(8) eV to 7124.0(8) eV near the iron-K absorption edge. At smaller scattering vector (*q*) values (*q* < 3 nm^–1^), where larger structures scatter, the signal mainly comes from the pores and pore walls in the carbon support, with no noticeable energy dependence, as shown in the zoomed-in inset of Figure 3a,b. At larger *q*-values (*q* > 3 nm^–1^), slight scattering differences are likely due to resonant Raman scattering, which dominates and obscures the expected scattering from small iron moieties. The absence of anomalous scattering effects (insets of Figure 3a,b) indicates that no iron-containing nanostructures larger than approximately 0.5 to 1 nm are present in either catalyst. This suggests that the iron is well distributed as single atomic iron centers, dimers, or small clusters. Therefore, the active iron centers are either (a) evenly distributed within the carbon support or (b) integrated into the carbon structure rather than existing in a metallic state, and we are dealing with mixed phases and oxidized iron forms, as confirmed by XANES.

**Figure 3:**
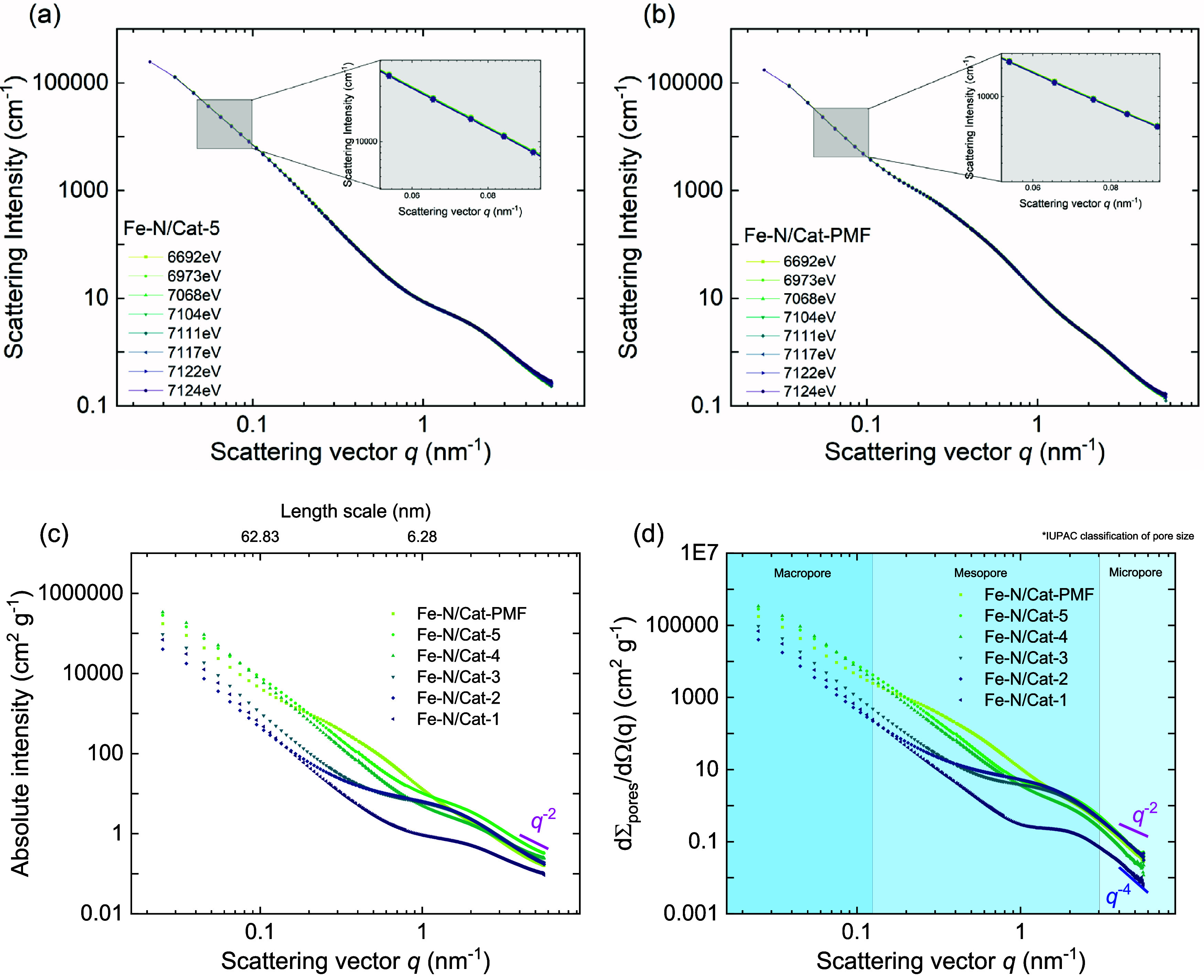
(a, b) Collected SAXS intensities as a function of the scattering vector (*q*) at various X-ray energies, ranging from 6692.0(8) eV to 7124.0(8) eV: (a) Fe–N/Cat-5 electrocatalyst and (b) Fe–N/Cat-PMF electrocatalyst. The enlarged view of the highlighted regions demonstrates the absence of anomalous scattering effects indicating a lack of iron nanostructures >1 nm. (c, d) Structural model-free SAXS analysis. (c) Mass-normalized scattering curves of Fe–N/Cat-X powders compared with commercial Fe–N/Cat-PMF at X-ray energy 7124.0(8) eV. A length scale is calculated by 2π/*q* and shown on the upper *x*-axis. (d) The fluctuation-induced contributions (eqs S1–S3) have been subtracted from the measured scattering intensities (c). As a result, the scattering patterns (with boundaries defined according to IUPAC classifications of porosity) presented in this graph solely reflect the pore structure of a two-phase system. For all samples, the characteristic *q* ^–4^ slope was recovered at high scattering vectors (eq S4).

This subsection describes a structural model-free SAXS analysis for studying six Fe–N/Cat-X electrocatalysts (Table 2). The effect of pore modifier (ZnCl_2_), use of a hard template (HA), and change of the pyrolysis temperature (800 °C vs 1000 °C) on the nanoscopic properties of the Fe–N/Cat-X catalysts within the length scale from 1.25 to 250 nm is observed (Figure 3c,d). The SAXS structural model-free analysis approach introduced by Ruland and co-workers^36–39^ and demonstrated on several occasions^40–44^ (eqs S1–S10) consists of separating the scattering intensity per unit mass of carbon into the scattering originating from the porous structure of the carbon $\frac{\underset{\mathrm{pores}}{d\sum }}{d\Omega }$ and the scattering from the density fluctuations of the carbon material $\frac{\underset{\mathrm{fluct}}{d\sum }}{d\Omega }$ (eqs S1–S3). After considering the fluctuation component, the rise of the pore scattering curve in the high scattering vector (*q*) region with a slope corresponding to *q* ^–4^ is related to an ideal two-phase structure (pores and homogeneous carbon phase) (eq S4 and Figure S6). Using a structural model-free approach, 13 structurally significant, mathematically independent, and physically meaningful parameters have been determined to characterize this study’s disordered microporous carbon-based **Fe–N/Cat-X** catalysts (Tables 3 and S2). The following parameters have been calculated from raw SAXS data (Figure 3c,d), namely, background scattering, *C*; fluctuation component, *B*_fl_; Porod constant, *P*_m_; internal surface area per mass, *S*/*m*, and Ruland length (length of the graphene layer curvature**)**, *l*_R_ (eqs S2–S4). Further determined parameters are porosity-related, such as invariant *Q* (*Q*_m_), porosity (ϕ), Porod length (*l*_p_), average chord length of pores (*l*_pore_), average chord length of pore walls (*l*_solid_), average chord length (*l*_c_), anisometric ratio (*l*_c_/*l*_p_), and degree of disorder (DoD) ( $\frac{\Delta^{2}a_{3}}{a_{3}^{2}}+\frac{\Delta^{2}l_{R}}{l_{R}^{2}}$), according to eqs S5–S10. The following subsections will analyze the determined scattering intensities of an ideal two-phase system $\frac{\underset{\mathrm{pores}}{d\sum }}{d\Omega }$ as a function of the scattering vector (*q*) for **Fe–N/Cat-X** electrocatalysts and discuss the newly obtained SAXS results (Tables 3 and S2) in empirical correlation with electrochemical characteristics.

**Table 3:** Selected Structural Parameters Are Derived from SAXS Analysis^a^

parameter	Fe–N/Cat-1	Fe–N/Cat-2	Fe–N/Cat-3	Fe–N/Cat-4	Fe–N/Cat-5	Fe–N/Cat-PMF
*C* (cm^2^g^–1^)	0.051 ± 0.002	0.038 ± 0.003	0.04 ± 0.004	0.096 ± 0.002	0.646 ± 0.003	0.062 ± 0.002
*S*/*m* (m^2^ g^–1^)	36.7 ± 7.3	193.0 ± 22.2	196.0 ± 22.6	100.9 ± 13.1	214.7 ± 23.9	168.5 ± 18.5
*l*_R_ (nm)	1.7	1.4	2.3	1.3	3.0	7.0
ϕ	0.03 ± 0.007	0.10 ± 0.009	0.10 ± 0.01	0.14 ± 0.001	0.18 ± 0.001	0.19 ± 0.001
*l*_pore_ (nm)	1.79 ± 1.07	1.22 ± 0.14	1.15 ± 0.170	4.04 ± 1.27	2.13 ± 0.28	2.71 ± 0.35
*l*_solid_ (nm)	59.2 ± 22.4	10.50 ± 1.16	10.04 ± 1.40	24.86 ± 2.83	9.4 ± 0.9	11.71 ± 0.95
$\frac{\Delta^{2}a_{3}}{a_{3}^{2}}+\frac{\Delta^{2}l_{R}}{l_{R}^{2}}$	0.04 ± 0.015	0.107 ± 0.012	0.111 ± 0.016	0.14 ± 0.015	0.22 ± 0.02	0.07 ± 0.006

a C—background scattering*; S/m*—internal surface area per mass; *l*_R_—length of the graphene layer curvature; ϕ—porosity; *l*_pore_—average chord length of pores; *l*_solid_—average chord length of pore walls; $\frac{\Delta^{2}a_{3}}{a_{3}^{2}}+\frac{\Delta^{2}l_{R}}{l_{R}^{2}}$ —degree of disorder (DoD).

### Impact of ZnCl_2_ as a Pore Modifier on Structure–Property Relationships

The diamond-shaped diagram with **Fe–N/Cat-1** at the top is connected to **Fe–N/Cat-2** via twice the amount of a pore modifier (ZnCl_2_), and **Fe–N/Cat-2** is in turn connected to **Fe–N/Cat-3** via pyrolysis temperatures (Figure 1a). SEM analysis of the three iron–nitrogen–carbon catalyst variants (**Fe–N/Cat-1**, **Fe–N/Cat-2**, and **Fe–N/Cat-3**) revealed predominantly large carbon grains. These catalysts demonstrated significant microporosity, contributing to their high specific surface areas of approximately 1000 m^2^ g^–1^ (Table 2). Previous research indicated that the ZnCl_2_ quantity affected mesoporosity development: **Fe–N/Cat-1** showed almost no mesoporosity, while **Fe–N/Cat-2** and **Fe–N/Cat-3** exhibited moderate mesoporosity. Mercury porosimetry confirmed limited meso- and macroporosity in the catalysts synthesized using only ZnCl_2_. Increasing pyrolysis temperature from 800 to 1000 °C in **Fe–N/Cat-3** resulted in reduced porosity between 100 and 2000 nm.^11^ The graph of the scattering patterns is conceptually divided into three regions with boundaries defined according to the IUPAC classifications of porosity (Figure 4a,b).

**Figure 4:**
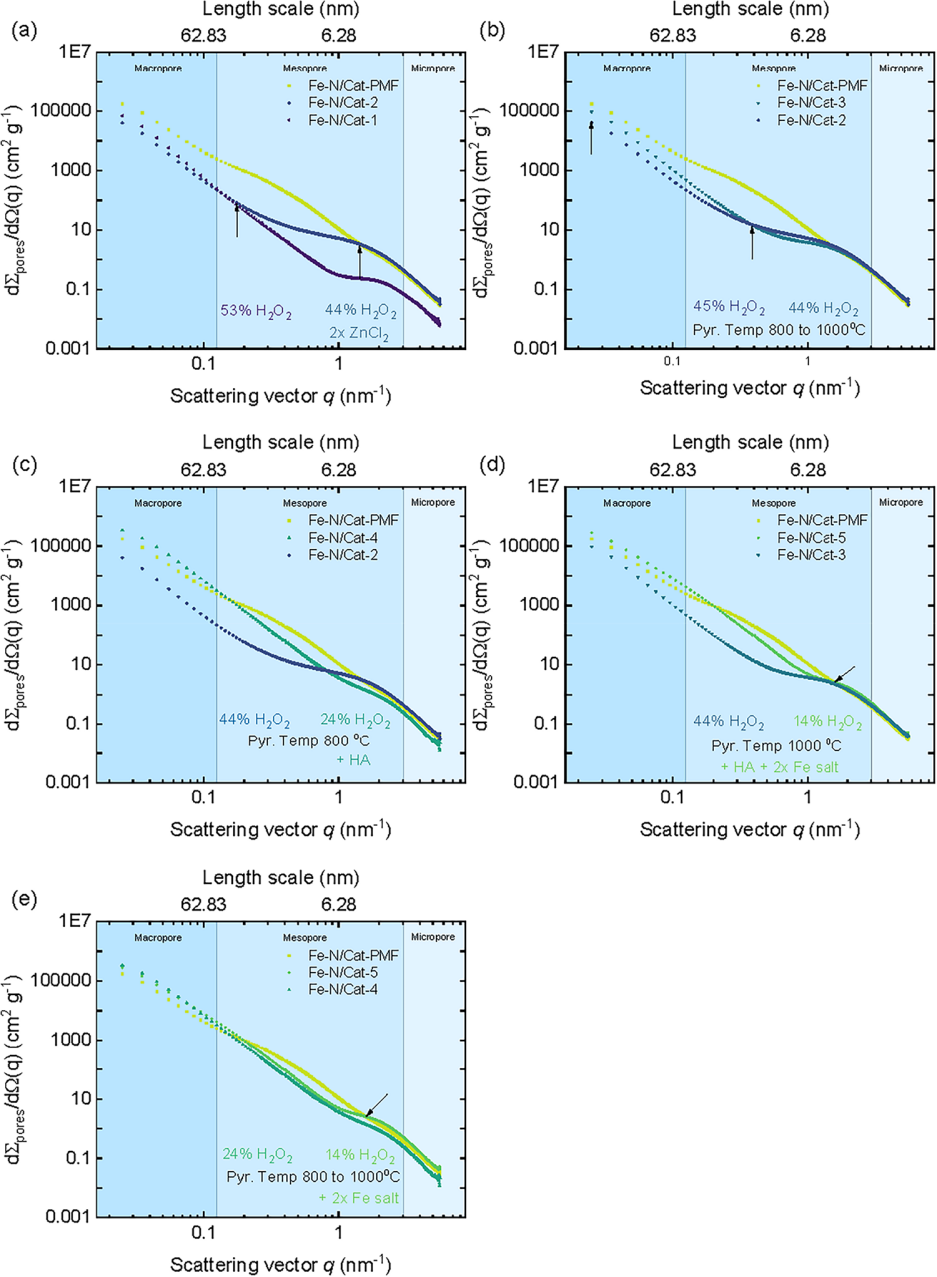
(a, b) Pore modifier (ZnCl_2_) induced structural changes. (a) A 2-fold increase in the ZnCl_2_ content during electrocatalyst synthesis significantly enhances mesopore and micropore formation. (b) Increasing the pyrolysis temperature from 800 to 1000 °C induces structural changes, primarily affecting pores up to 36 nm and larger within the macropore range. (c, d) The strong templating effect of HA on pore architecture. (c) Introducing a hydroxyapatite induces pronounced structural transformations across the full hierarchical pore scale, significantly altering meso- and macropore structures while promoting micropore merging at a pyrolysis temperature of 800 °C. (d) Structural changes in the hierarchical pore structure, including the introduction of a hard template and the doublet of the iron salt content at a pyrolysis temperature of 1000 °C. (e) A comparison of Fe–N/Cat-4, Fe–N/Cat-5, and Fe–N/Cat-PMF (included as a benchmark), highlighting the impact of increased pyrolysis temperature and combined use of ZnCl_2_ and HA in Fe–N/Cat-5 synthesis. These conditions enhanced the hierarchical structure, especially in the micropore and mesopore regions, and improved the electrochemical performance, resulting in a lower peroxide yield.

Figure 4a shows that doubling the amount of pore modifier ZnCl_2_ significantly enhances mesopore formation in the 6.3 nm range, as evidenced by an increase in pore scattering intensity by more than an order of magnitude (**Fe–N/Cat-1** to **Fe–N/Cat-2**). Increasing the ZnCl_2_ content also affects the pore scattering intensity in the micropore range. Interestingly, while the average mesopore size increases, the larger pores, including those up to 36 nm and beyond in the macropore range, remain unaffected by ZnCl_2_. SEM-EDS compositional analysis confirmed the presence of Fe, N, C, and O in **Fe–N/Cat-1** and **Fe–N/Cat-2**, with residual Zn from the precursor. **Fe–N/Cat-3** exhibited reduced N and Zn content due to higher pyrolysis temperatures, a typical outcome for N- and Zn-containing catalyst precursors.^45,46^ Extensive structural changes can be observed via structural model-free SAXS analysis in the material at every level (Table 3), affecting key parameters such as *S*/*m* (internal surface area per mass), ϕ (porosity), *l*_pore_ (average chord length of pores), *l*_solid_ (average chord length of pore walls), and $\frac{\Delta^{2}a_{3}}{a_{3}^{2}}+\frac{\Delta^{2}l_{R}}{l_{R}^{2}}$ (degree of disorder (DoD)). The ORR activity and selectivity were evaluated in 0.1 M KOH by using the rotating ring-disk electrode (RRDE) method. Doubling the ZnCl_2_ content in the **Fe–N/Cat-2** synthesis improved the ORR activity, increasing the onset potential (*E*_onset_) value from 0.90 V vs RHE (**Fe–N/Cat-1**) to 0.92 V vs RHE. Additionally, hydrogen peroxide yield (*x* H_2_O_2_ at 0.5 V vs RHE) in **Fe–N/Cat-2** decreased by approximately 17% compared to **Fe–N/Cat-1** (Table 2). The electrochemical results are supported by the following structural changes: a significant increase in *S*/*m* (approximately 5.5 ± 0.2 times) and a corresponding decrease in the *l*_pore_ by the same factor compared to those of **Fe–N/Cat-1**. These changes, along with the formation of new mesopores and micropores at the expense of micropore walls and an increase in structural disorder, indicating reduced graphitization, further validate the electrochemical findings. Increasing the pyrolysis temperature from 800 to 1000 °C enhanced the onset potential of **Fe–N/Cat-3** by 20 mV. This improvement is likely linked to structural modifications, including changes in pore walls, e.g., length of the graphene layer curvature (*l*_R_), and the Porod length (*l*_p_) of larger pores, particularly those up to 36 nm and beyond in the macropore range (Figure 4b). These changes, reflected in the increased average chord length (*l*_c_) and anisometric ratio (*l*_c_/*l*_p_), contribute to enhanced diffusion within the **Fe–N/Cat-3** catalyst (Table S2). The transition to a more slit-shaped pore structure further supports this effect. Despite these structural adjustments, the hydrogen peroxide production rate remained nearly unchanged, with **Fe–N/Cat-3** at 45% and **Fe–N/Cat-2** at 44% (Table 2), indicating minimal temperature influence on this parameter. Moreover, **Fe–N/Cat-2** and **Fe–N/Cat-3** exhibit similarities across nearly all structural characteristics, including *l*_pore_, *l*_solid_, and *S*/*m*. Consequently, the availability of reaction centers for the further conversion of H_2_O_2_ to H_2_O on the pore surface remains limited in both catalysts at a selected potential of 0.5 V vs RHE.

### Impact of Hydroxyapatite as a Hard Template on Structure–Property Relationships

The diamond-shaped diagram illustrates how **Fe–N/Cat-2** branches into two distinct catalyst variants, **Fe–N/Cat-3** and **Fe–N/Cat-4**, depending on the synthesis precursor (Figure 1a). **Fe–N/Cat-3**, shown on the right, was synthesized at a pyrolysis temperature of 1000 °C, as discussed in a previous section. On the left, **Fe–N/Cat-4** was synthesized at 800 °C with the addition of a hard template, hydroxyapatite. In this subsection, we will examine the impact of HA addition. Incorporating HA as a needle-like powder with a particle size of (60 ± 10) nm in the synthesis mixture of **Fe–N/Cat-4** resulted in a complete transformation of the material’s morphology, producing a powder with significantly smaller particles. Thus, the inclusion of HA as a hard template led to a substantial increase in mesoporosity, as confirmed by the N_2_ sorption method (Table 2). Despite the pronounced structural modifications, the addition of HA had minimal impact on the *E*_onset_ value, while in the case of **Fe–N/Cat-4**, the hydrogen peroxide yield dropped significantly, decreasing by 43% compared to **Fe–N/Cat-2**. Using a hard template (HA) induces significant structural changes across the entire hierarchical length scale observed for **Fe–N/Cat-4** (Figure 4c,d and Table 3), affecting key parameters such as *S*/*m*, *l*_pore_, and *l*_solid_. HA notably impacts mesopores and macropores while also merging micropores. The pronounced increase in the intensity of macro- and mesopores suggests a substantial rise in the transport pores and reaction centers. This structural evolution, characterized by an increase in mesopore scattering and their hierarchical integration into macropores, appears to facilitate H_2_O_2_ reduction. The structure exhibits an increased pore wall thickness, indicating that precursors become trapped within smaller pores. Previous SEM-EDS analysis revealed that **Fe–N/Cat-4** contains significantly higher Fe and Zn levels, along with residual Ca and P from HA, suggesting that these elements are not fully removed during acid treatment (Table 2).

Another way to assess the effect of HA is to compare the catalysts **Fe–N/Cat-3** and **Fe–N/Cat-5** (Figure 4d), and it is important to note that both samples were subjected to the same pyrolysis temperature, and both exhibit relatively low levels of impurities (Table 2). According to the diamond-shaped diagram (Figure 1a), these catalysts differ in the amount of Fe and the addition of HA during the synthesis process, and in the case of these catalysts, the amount of residuals in the final catalyst is significantly lower. According to the results of the SEM-EDS analysis, these materials, pyrolyzed at higher temperatures, do not contain Zn or Ca, and there is only a small P residual. To enable quantitative SAXS analysis, we used SEM-EDS elemental composition data (Table 2) as input to calculate the scattering contrast of each electrocatalyst (eq S7). **Fe–N/Cat-5** demonstrated the highest activity among all tested catalysts, with an onset potential of 0.96 V vs RHE and a hydrogen peroxide yield of 14%, representing a 68% reduction in peroxide yield compared to **Fe–N/Cat-3**, where ZnCl_2_ was the only pore-modifying agent. This strong structure–property relationship underlies the exceptional efficiency of **Fe–N/Cat-5**. A comparison of the results of SAXS analysis reveals that the structural shift mainly affected the mesopore and macropore fractions, since the scattering vector (*q*) was larger than 1. At the same time, the pore scattering contribution of the micropores remained the same. When comparing the SAXS patterns for **Fe–N/Cat-3** and **Fe–N/Cat-5** and for **Fe–N/Cat-4** and **Fe–N/Cat-5**, this controlled consistency allows us to take into account the effects of residuals and structural features on the resulting pore distribution and lateral imperfections, i.e., *q*-values for electrocatalysts deviates from the value of −4 indicating surface roughness and possible pore blockage consistent with residuals. (Figure 3c). Thus, the further quantitative analysis via structural model-free approach was carried out (Figure S6) of the SAXS data provides direct evidence that residuals somewhat impact microporosity (Figure 3d). The **Fe–N/Cat-4** sample, which contains a higher level of residuals and has mixed phases and oxidized iron (Fe_2_O_3_) according to **XANES** analysis, displays a noticeably lower SAXS intensity than that of **Fe–N/Cat-3**. This decrease in intensity indicates a diminished microporous structure, supporting the conclusion that residuals hinder the development or preservation of micropores during the synthesis process.

At the bottom of the diamond-shaped diagram (Figure 1a), **Fe–N/Cat-5** represents a combination of different synthesis conditions, including the use of a pore modifier (ZnCl_2_) and a hard template (HA), doubling the amount of iron salt, and increasing the pyrolysis temperature from 800 to 1000 °C, which led to suitable hierarchical porosity and the best electrochemical performance. A comparison between **Fe–N/Cat-4** and **Fe–N/Cat-5**, both synthesized with HA, shows that the higher pyrolysis temperature used for **Fe–N/Cat-5** reduced the phosphorus content, as indicated by **SEM-EDS** analysis (Table 2). The elevated temperature facilitated the complete removal of Zn precursor residues, preventing Zn from being trapped in the catalyst powder matrix, a trend also observed in catalysts where ZnCl_2_ was the sole pore modifier. Favorably, the combination of HA usage and the increased pyrolysis temperature used to synthesize the **Fe–N/Cat-5** electrocatalyst led to significant structural modifications that directly influenced its catalytic performance. While HA facilitated the formation of essential transport pores, the elevated temperature promoted the generation of accessible mesopores within the pore walls, reducing the pore wall thickness (*l*_solid_) in the **Fe–N/Cat-5** catalyst. This structural shift primarily affected the micropore and mesopore regions, while the intensity of the macropores remained largely unchanged (Figure 4e). Additionally, doubling the iron salt content introduced further structural and compositional changes, namely, (a) the formation of locally trapped Fe_2_P compounds, which acid treatment did not remove entirely,^11^ contributing to SAXS analysis as an increased background scattering (eq S2), and (b) the incorporation of Fe into the carbon matrix, as confirmed by ASAXS analysis. The intracarbon matrix Fe moieties are uniformly distributed and nonmetallic, contributing to expanded reaction sites at the accessible internal surface area, favoring fast H_2_O_2_ decomposition. The hierarchical opening of mesopores (enhanced by the needle-like HA) into macropores further improved catalytic efficiency by enhancing reactant/product accessibility. Consequently, the hierarchical porosity improved diffusion pathways, and additional temperature increases enhanced the number of reaction centers, aligning with the electrochemical results. Thus, in the study of a series of materials designated **Fe–N/Cat-1** through **Fe–N/Cat-5**, these structural enhancements translated into superior catalytic performance of **Fe–N/Cat-5** (*E*_onset_ = 0.96 V vs RHE, H_2_O_2_ yield = 14%).

A notable observation is a clear empirical correlation between the *l*_pore_/*l*_solid_ ratio, degree of disorder (DoD), and the H_2_O_2_ yield and the *E*_onset_, reflecting structural evolution as their values gradually increase across the electrocatalyst series. Interestingly, in the cases of **Fe–N/Cat-2** and **Fe–N/Cat-3**, a plateau effect is observed: DoD and the *l*_pore_/*l*_solid_ ratio remain relatively constant, which coincides with a stable H_2_O_2_ yield (%) (Figure 5a) and number of electrons transferred per molecule of oxygen (*n*) (Table 2).

**Figure 5:**
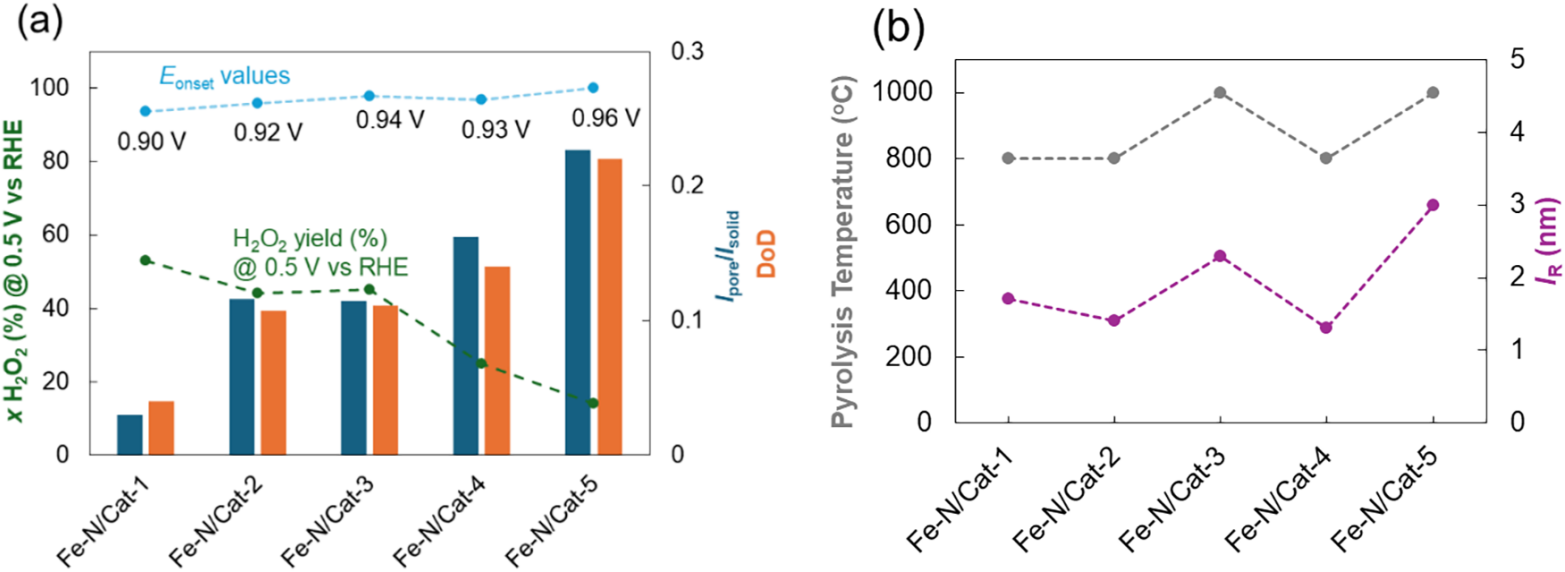
Empirical correlations between structural and electrochemical parameters. (a) Both, the *l*_pore_/*l*_solid_ ratio and DoD parameters (columns) consistently trend with *E*_onset_ value—showing a gradual increase across the electrocatalyst series. For Fe–N/Cat-2 and Fe–N/Cat-3, the DoD and *l*_pore_/*l*_solid_ values plateau parallel with the H_2_O_2_ yield (%) at 0.5 V vs RHE. Among the evaluated electrocatalysts, Fe–N/Cat-5 demonstrates the most advantageous combination of a high onset potential (*E*_onset_), low H_2_O_2_ yield, elevated *l*_pore_/*l*_solid_ ratio, and enhanced degree of disorder (DoD) structural parameter, collectively indicating its superior capability to promote the selective 2e^–^ + 2e^–^ oxygen reduction reaction (ORR) pathway. (b) The *l*_R_ parameter is prominently influenced by the pyrolysis temperature.

Figure 5b demonstrates that the *l*_R_ values are more pronounced in electrocatalysts synthesized at higher pyrolysis temperatures, indicating an enhanced curvature. This structural evolution is especially significant in **Fe–N/Cat-5**, where the less curved graphene layers contribute to a higher *E*_onset_ and facilitate a favorable 2e^–^ + 2e^–^ oxygen reduction reaction pathway.

Our observations demonstrate that higher pyrolysis temperatures lead simultaneously to an increase in the structural parameter *l*_R_, accompanied by a decrease in the nitrogen and zinc contents in the catalyst. On one hand, the zinc evaporation promotes the formation of a porous carbon structure while preserving the desired iron–nitrogen active sites, thereby enhancing ORR performance. On the other hand, increasing the pyrolysis temperature alters the nitrogen content and configuration of the electrocatalyst, as reported previously.^45^ XPS analysis with elemental qualification indicates a decrease in the total nitrogen content due to the loss of pyridinic N and the gain of Fe–N_*x*_ sites (Table S1). The correlation between the ORR selectivity, total nitrogen content, and proportion of the Fe–N_*x*_ sites and structural parameter *l*_R_ for selected electrocatalysts, including **Fe–N/Cat-PMF**, is summarized in Figure 6. This suggests that the ORR performance of **Fe–N/Cat-5** primarily depends on the synthesis conditions, which facilitate the formation of favorable active sites and an optimized porosity through the use of a hard hydroxyapatite template confirming the reported correlation between ORR performance and the proportion of Fe–N_*x*_ and graphitic compounds.

**Figure 6:**
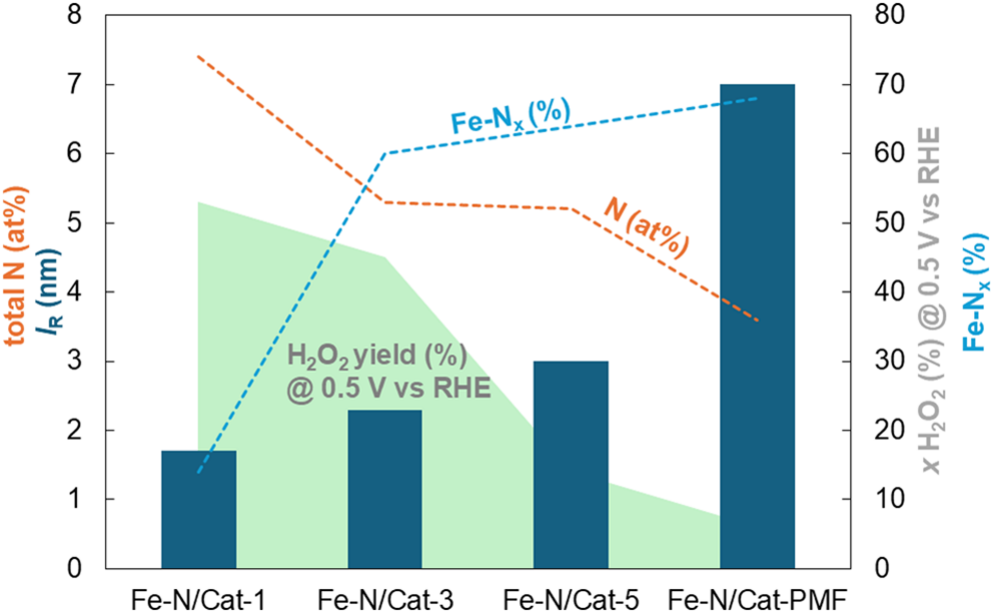
Correlation between the ORR performance (H_2_O_2_ yield % at 0.5 V vs RHE)—area, XPS analysis (total nitrogen amount and proportion of Fe–N_*x*_)—dashed lines, and SAXS structural parameter (length of the graphene layer curvature (*l*_R_))—columns.

Structural analysis reveals that compared to the studied commercial **Fe–N/Cat-PMF** electrocatalyst, which exhibited a lower peroxide yield, the **Fe–N/Cat-5** material demonstrates significant advancements in key morphological features. Notably, it possesses an optimized average pore width, enhanced pore wall thickness, and most critically, the length of the graphene layer curvature (*l*_R_) of the pore wall. The parameter *l*_R_ describes the correlation length in the plane of the carbon rings, which is finite due to the bending and finite sizes of the carbon patches. This length scale is independent of the stacking of carbon layers that form the pores and is determined by the bending stiffness of the carbon layers. Thus, a higher *l*_R_ value indicates less curved graphene layers. Moreover, the length of the graphene layer curvature plays a crucial role in spatially arranging multiple active centers at optimal distances from one another. Such an arrangement promotes efficient electron and intermediate transfer, thereby facilitating a highly effective and selective ORR 4-electron pathway. Structural model-free SAXS analysis demonstrates that a step-by-step approach to modifying synthesis conditions allowed us to achieve structural properties closely resembling those of the commercially produced catalyst **Fe–N/Cat-PMF** (Figure 4e). Structural parameters, including average graphene layer curvature—Ruland length (*l*_R_), porosity (ϕ), average chord length of pores (*l*_pore_), average chord length of pore walls (*l*_solid_), and degree of disorder (DoD) contributing to the best-performing, environmentally friendly **Fe–N/Cat-5** catalyst (Table 3).

To further enhance the catalytic performance of **Fe–N/Cat-5**, additional optimization is needed, particularly to tune mesoporosity (Figure 4e) and the form of nitrogen (Fe–N_*x*_ and graphitic nitrogen). In the literature, active catalysts have been obtained at a pyrolysis temperature of 1100 °C due to the favorable combination of active components and a well-developed carbon matrix.^45^ Qu et al.^47^ found that the catalyst material synthesized at a pyrolysis temperature of 1100 °C exhibits better durability and reliability, as well as stable formation of Fe–N_*x*_ centers. The **Fe–N/Cat-5** electrocatalyst, synthesized at a pyrolysis temperature of 1000 °C, demonstrates in the accelerated tests over 10,000 cycles that the *E*_onset_ value decreases approximately 20 mV (Figure S1). We can conclude that further increasing the pyrolysis temperature above 1100 °C does not necessarily yield more active materials but may improve their stability. Therefore, choosing the optimal pyrolysis temperature is an important step in ensuring a good balance among the activity, stability, and selectivity.

Key factors include the selection of pore modifiers and hard templates and the specific stage at which higher pyrolysis temperatures are introduced. This step-by-step analysis suggests that pore curvature is a vital design parameter among others, and future synthesis strategies should deliberately aim to tailor the mesoporous structure, particularly its curvature, to enhance the catalytic performance.

## Conclusions

Understanding the correlation between the structure of catalyst materials and the electrochemical activity and selectivity is crucial for developing hydrogen energy and fuel cell technology. For practical AEMFC catalysts, it is assumed that the peroxide yield is less than 5% in the electrode potential range of 0.5 to 0.8 V. Using hydroxyapatite as a hard template in addition to the pore modifier ZnCl_2_, a hierarchically porous (micro-, meso-, and macroporous) Fe–N–C electrocatalyst with very high ORR activity and selectivity in alkaline media was obtained. This study demonstrates the benefits of anomalous and small-angle X-ray scattering (ASAXS/SAXS) in monitoring the progressive development of micro- and mesoporosity during the synthesis of Fe–N–C electrocatalysts. Comprehensive structural model-free SAXS analysis demonstrates the importance of employing advanced characterization techniques to more accurately evaluate disordered carbonaceous material-based electrocatalysts. Thirteen SAXS-derived structural parameters were simultaneously obtained for five Fe–N–C electrocatalysts and benchmarked against those of the commercial Fe–N/Cat-PMF material. Comparing Fe–N/Cat-X prepared at different synthesis conditions shows that parameters such as average graphene layer curvature (*l*_R_), porosity (ϕ), ratio of average chord length of pores (*l*_pore_) to average chord length of pore walls (*l*_solid_), and degree of disorder (DoD) increase step by step, contributing to the high performance of the environmentally friendly Fe–N/Cat-5 electrocatalyst. The most important of these is the *l*_R_, which describes how active iron moieties are distributed (separated) and features a “pore-wall-specific” curvature that enhances the ORR activity of the catalyst and accelerates the selectivity of the 2e^–^ + 2e^–^ ORR reaction pathway. Empirical correlations showed that optimizing structural parameters, such as *l*_R_, *l*_pore_/*l*_solid_, and DoD, supports higher *E*_onset_ and lower H_2_O_2_ yield (%). We suggest an average graphene layer curvature of at least 3 nm to enhance the selectivity and boost the 2e^–^ + 2e^–^ ORR reaction pathway. These findings support the idea that future synthesis strategies should aim to modify the pore curvature and form a mesoporous structure to improve catalytic ORR performance.

## Methods

### Anomalous Small-Angle X-ray Scattering

ASAXS measurements of **Fe–N/Cat-X** electrocatalysts were conducted at the four-crystal monochromator (FCM) beamline of the PTB using the HZB-ASAXS instrument at the BESSY II synchrotron radiation facility, operated by the Helmholtz-Zentrum Berlin, Germany.^48^ ASAXS measurements were conducted in transmission mode under ex situ conditions. In a typical ASAXS measurement, a series of scattering patterns are measured at different X-ray energies near and, in most cases, below the absorption edge of the element of interest at which its anomalous scattering coefficients and, with that, the effective electron density ρ significantly changes. These measurements encompassed six **Fe–N/Cat-X** electrocatalyst powders, and experiments utilized a photon energy range between 6692.0 and 7124.0 eV (Δ*E*/*E* ∼ 2 × 10^–4^), near the K absorption edge of Fe (7111 eV measured, 7112 eV theory for pure Fe). ASAXS data were captured using a specialized in-vacuum Pilatus 1 M detector (Dectris Ltd., Baden, Switzerland) at a sample-to-detector distance of (0.810 ± 0.005) m and (3.779 ± 0.005) m.^49^ Thus, this covers a scattering vector *q*-range from about 0.025 nm^–1^ to 5.65 nm^–1^. The samples were measured with an exposure time of 60 s and four and six repetitions for shorter and longer sample-to-detector distances, respectively, to achieve good-quality data and monitor unwanted but possible changes in the sample over time. The sample detector distance was determined via the triangulation method based on the long-period spacing *d*001 peak of the silver behenate (CH_3_(CH_2_)_20_COO·Ag).^50^ Initial experiments included measurements of the empty beam to assess the scattering contributions from the background. A glassy carbon standard was also used for absolute intensity calibration into differential scattering cross sections for all samples.^51^ Data processing and analysis were facilitated using the advanced version of “BerSAS” software,^52^ with background scattering intensities measured independently under identical conditions and subtracted from the scattering intensities of the samples. The data were adjusted for the sample thickness and transmission values. The 2D ASAXS data were radially averaged to generate 1D scattering profiles—intensity vs scattering vector *q*.

In the case of anomalous scattering, the atomic scattering factor is given as a complex: *f* = *f*_0_ + *f*′ + i*f*″, where *f*_0_ is the short wavelength limit of the scattering amplitude, e.g., *f*_0_(0) of iron is 26, that is, the number of electrons (Figure S5).^53^ While the variation of (*f*_0_ + *f*′) is small away from the edge, it becomes strong and nonlinear near the edge (Figure S5). The theoretical values of the scattering factors were calculated using the procedure described by Cromer and Libermann.^54,55^ Dependence of the real part (*f*_0_ + *f*′) on the X-ray energy for iron and carbon elements with absorption edges in the range 6000–7150 eV around the Fe K-edge.^56^

### X-ray Fluorescence and X-ray Absorption Near-Edge Structure

For the XRF measurements, the samples were mounted on an aluminum frame and installed in a sample chamber on a motorized sample stage with six degrees of freedom^57^ attached to the FCM beamline.^48^ The X-ray beam was collimated to approximately 0.5 × 0.5 mm^2^ and focused on the samples at an angle of incidence of 10° relative to the surface normal. Fluorescence radiation was collected using a silicon drift detector (XFlash 6|20, Bruker Nano, Berlin, Germany) at an angle of 35°. The photon energy of the beam was set to 10 keV, resulting in a photon flux of ∼10^9^ s^–1^. The electrocatalysts were fixed between two sheets of sticky tape, alternately moved into the beam, and illuminated for 900 s at 10 keV while collecting the fluorescent radiation.

### Structural Parameters—Small-Angle X-ray Scattering

Small-angle X-ray scattering (SAXS) is an elastic scattering method carried out in transmission geometry, which uses diffuse scattering around the transmitted beam to investigate structures with sizes larger than interatomic distances to about 100 nm in a sample.^58^ The scattering intensity is proportional to the second power of the electron density difference between nanosized structures and their surroundings, known as scattering contrast, within the sample.^59^ SAXS 1D scattering profiles were analyzed via structural model-free analysis using a combination of Schiller, Mering, Perret, and Ruland approximations (SI, Figure S6).^36–39^ This approach identifies up to 13 structurally significant, mathematically independent, and physically meaningful parameters, serving as a valuable toolbox for understanding disordered micromesoporous carbonaceous materials.^40–44^ The structural parameters, such as the average chord length of pores (*l*_pore_), the average chord length of pore walls (*l*_solid_), the degree of disorder (DoD), internal surface area per mass (*S*/*m*), and the curvature of the graphene layer (*l*_R_), give a detailed description of the disordered carbonaceous material.^40–44^

## Supplementary Material



## Data Availability

Requests for the ASAXS/SAXS data and analysis utilized in this work will be handled by the lead contact, Eneli Härk (eneli.monerjan@helmholtz-berlin.de). Requests for the electrochemical data utilized in this work will be handled by Rutha Jäger (rutha.jager@ut.ee).

## Notes

Additional information on the chemicals and materials used for the Fe–N/Cat-X synthesis; physical characterization (SEM, low-temperature N_2_ sorption, TEM, XRD, XPS, theoretical scattering factors calculated using the Cromer and Libermann procedure, and structural model-free SAXS data analysis including the separation of fluctuation and ideal two-phase components); electrochemical evaluation via RRDE; and derived structural parameters via SAXS (PDF)

## References

[1] Manufacturing Cost Analysis of 100 and 250 kW Fuel Cell Systems for Primary Power and Combined Heat and Power Applications 2016 https://www.energy.gov/eere/fuelcells/articles/manufacturing-cost-analysis-100-and-250-kw-fuel-cell-systems-primary-power.

[2] Path to hydrogen competitiveness: A cost perspective 2020 https://hydrogencouncil.com/en/path-to-hydrogen-competitiveness-a-cost-perspective/.

[3] Reverdiau G., Le Duigou A., Alleau T., Aribart T., Dugast C., Priem T. (2021). Will there
be enough platinum for a large deployment of fuel cell
electric vehicles?. Int. J. Hydrogen Energy.

[4] Hossen M. M., Hasan M. S., Sardar M. R. I., Haider Jb., Mottakin, Tammeveski K., Atanassov P. (2023). State-of-the-art and developmental trends in platinum
group metal-free cathode catalyst for anion exchange membrane fuel
cell (AEMFC). Appl. Catal, B.

[5] Asset T., Atanassov P. (2020). Iron-Nitrogen-Carbon Catalysts for
Proton Exchange
Membrane Fuel Cells. Joule.

[6] Serov A., Artyushkova K., Atanassov P. (2014). Fe-N-C Oxygen Reduction Fuel Cell
Catalyst Derived from Carbendazim: Synthesis, Structure, and Reactivity. Adv. Energy Mater..

[7] Osmieri L., Escudero-Cid R., Videla A. H. A. M., Ocón P., Specchia S. (2017). Performance of a Fe-N-C
catalyst for the oxygen reduction
reaction in direct methanol fuel cell: Cathode formulation optimization
and short-term durability. Appl. Catal., B.

[8] Li S., Cheng C., Zhao X., Schmidt J., Thomas A. (2018). Active Salt/Silica-Templated
2D Mesoporous FeCo-N­(x) -Carbon as Bifunctional Oxygen Electrodes
for Zinc-Air Batteries. Angew. Chem., Int. Ed..

[9] Huang Y., Chen Y., Xu M., Asset T., Tieu P., Gili A., Kulkarni D., De Andrade V., De Carlo F., Barnard H. S. (2021). Catalysts by pyrolysis:
Direct observation of chemical and morphological transformations leading
to transition metal-nitrogen-carbon materials. Mater. Today.

[10] Hua Y., Jiang T., Wang K., Wu M., Song S., Wang Y., Tsiakaras P. (2016). Efficient Pt-free electrocatalyst
for oxygen reduction reaction: Highly ordered mesoporous N and S co-doped
carbon with saccharin as single-source molecular precursor. Appl. Catal., B.

[11] Teppor P., Jäger R., Koppel M., Volobujeva O., Palm R., Månsson M., Härk E., Kochovski Z., Aruväli J., Kooser K. (2024). Unlocking
the porosity of Fe–N–C catalysts using hydroxyapatite
as a hard template en route to eco-friendly high-performance AEMFCs. J. Power Sources.

[12] Mineva T., Matanovic I., Atanassov P., Sougrati M.-T., Stievano L., Clémancey M., Kochem A., Latour J.-M., Jaouen F. (2019). Understanding
Active Sites in Pyrolyzed Fe–N–C Catalysts for Fuel
Cell Cathodes by Bridging Density Functional Theory Calculations and
57Fe Mössbauer Spectroscopy. ACS Catal..

[13] Matanovic I., Artyushkova K., Atanassov P. (2018). Understanding PGM-free catalysts
by linking density functional theory calculations and structural analysis:
Perspectives and challenges. Curr. Opin. Electrochem..

[14] Kramm U. I., Lefevre M., Larouche N., Schmeisser D., Dodelet J. P. (2014). Correlations between mass activity
and physicochemical
properties of Fe/N/C catalysts for the ORR in PEM fuel cell via 57Fe
Mossbauer spectroscopy and other techniques. J. Am. Chem. Soc..

[15] Chung H. T., Cullen D. A., Higgins D., Sneed B. T., Holby E. F., More K. L., Zelenay P. (2017). Direct atomic-level insight into
the active sites of a high-performance PGM-free ORR catalyst. Science.

[16] Charreteur F., Jaouen F., Ruggeri S., Dodelet J.-P. (2008). Fe/N/C non-precious
catalysts for PEM fuel cells: Influence of the structural parameters
of pristine commercial carbon blacks on their activity for oxygen
reduction. Electrochim. Acta.

[17] Vogtt K., Goerigk G., Ballauff M., Gläser R., Dingenouts N. (2013). Anomalous small-angle x-ray scattering
from mesoporous
noble metal catalysts. Colloid Polym. Sci..

[18] Tuaev X., Rudi S., Petkov V., Hoell A., Strasser P. (2013). In situ study
of atomic structure transformations of Pt-Ni nanoparticle catalysts
during electrochemical potential cycling. ACS
Nano.

[19] Polizzi S., Riello P., Goerigk G., Benedetti A. (2002). Quantitative
investigations of supported metal catalysts by ASAXS. J. Synchrotron Radiat..

[20] Jeng U. S., Lai Y.-H., Sheu H.-S., Lee J.-F., Sun Y.-S., Chuang W.-T., Huang Y.-S., Liu D.-G. (2007). Anomalous small-
and wide-angle X-ray scattering and X-ray absorption spectroscopy
for Pt and Pt–Ru nanoparticles. J. Appl.
Crystallogr..

[21] Haas S., Zehl G., Dorbandt I., Manke I., Bogdanoff P., Fiechter S., Hoell A. (2010). Direct Accessing the
Nanostructure
of Carbon Supported Ru–Se Based Catalysts by ASAXS. J. Phys. Chem. C.

[22] Haubold H.-G., W X.
H. (1995). ASAXS studies of carbon
supported electrocatalysts. Nucl. Instrum. Methods
Phys. Res., Sect. B.

[23] Gilbert J. A., Kariuki N. N., Subbaraman R., Kropf A. J., Smith M. C., Holby E. F., Morgan D., Myers D. J. (2012). In situ anomalous
small-angle X-ray scattering studies of platinum nanoparticle fuel
cell electrocatalyst degradation. J. Am. Chem.
Soc..

[24] Georgieva J., Valova E., Mintsouli I., Sotiropoulos S., Tatchev D., Armyanov S., Hubin A., Dille J., Hoell A., Raghuwanshi V. (2015). Pt­(Ni) electrocatalysts
for methanol oxidation prepared by galvanic replacement on TiO2 and
TiO2–C powder supports. J. Electroanal.
Chem..

[25] Bóta A., Varga Z., Goerigk G. (2008). Structural Description of the Nickel
Part of a Raney-Type Catalyst by Using Anomalous Small-Angle X-ray
Scattering. J. Phys. Chem. C.

[26] Heilmann M., Prinz C., Bienert R., Wendt R., Kunkel B., Radnik J., Hoell A., Wohlrab S., Buzanich A. G., Emmerling F. (2022). Size-Tunable
Ni–Cu Core–Shell Nanoparticles—Structure,
Composition, and Catalytic Activity for the Reverse Water–Gas
Shift Reaction. Adv. Eng. Mater..

[27] Yang R., Dahn T. R., Dahn J. R. (2009). Fe–N–C
Oxygen-Reduction
Catalysts Supported on “Burned-Off” Activated Carbon. J. Electrochem. Soc..

[28] Mostoni S., Mirizzi L., Frigerio A., Zuccante G., Ferrara C., Muhyuddin M., D’Arienzo M., Fernanda Orsini S., Scotti R., Cosenza A. (2025). In-Situ HF Forming Agents
for Sustainable Manufacturing of Iron-Based Oxygen Reduction Reaction
Electrocatalysis Synthesized Through Sacrificial Support Method. ChemSusChem.

[29] Lyu X., Dileep N. P., Pushkar Y., Pupucevski M., Lattimer J., Colon-Mercado H., Ganesan P., Ryder M. R., Keum J. K., Cullen D. A. (2025). Enhancing durability
and activity toward oxygen evolution reaction using single-site Re-doped
NiFeOx catalysts at ampere-level. Chem. Eng.
J..

[30] Lyu X., Chang H.-M., Yu H., Kariuki N. N., Hyung
Park J., Myers D. J., Yang J., Zenyuk I. V., Serov A. (2025). Evaluation
of IrO2 catalysts doped with Ti and Nb at industrially relevant electrolyzer
conditions: A comprehensive study. Chem. Eng.
J..

[31] Chatterjee S., Peng X., Intikhab S., Zeng G., Kariuki N. N., Myers D. J., Danilovic N., Snyder J. (2021). Nanoporous Iridium
Nanosheets for Polymer Electrolyte Membrane Electrolysis. Adv. Energy Mater..

[32] Menga D., Low J. L., Li Y. S., Arcon I., Koyuturk B., Wagner F., Ruiz-Zepeda F., Gaberscek M., Paulus B., Fellinger T. P. (2021). Resolving
the Dilemma of Fe-N-C Catalysts
by the Selective Synthesis of Tetrapyrrolic Active Sites via an Imprinting
Strategy. J. Am. Chem. Soc..

[33] Scattering Length Density Calculator. https://www.ncnr.nist.gov/resources/sldcalc.html.

[34] Beckhoff B. (2008). Reference-free
X-ray spectrometry based on metrology using synchrotron radiation. J. Anal. At. Spectrom..

[35] Exafs Materials https://www.exafsmaterials.com/foil-catalogue.

[36] Schiller C., Mering J., Cornuault P., Du Chaffaut F. (1967). Defauts structuraux
dans les carbones graphitables effets des traitements thermiques—partie
II. Carbon.

[37] Ruland W., Smarsly B. (2002). X-ray scattering of
non-graphitic carbon: an improved
method of evaluation. J. Appl. Crystallogr..

[38] Ruland W. (1971). Small-angle
scattering of two-phase systems: determination and significance of
systematic deviations from Porod’s law. J. Appl. Crystallogr..

[39] Perret R., Ruland W. (1968). X-ray small-angle scattering
of non-graphitizable carbons. J. Appl. Crystallogr..

[40] Kalder L., Olgo A., Lührs J., Romann T., Härmas R., Aruväli J., Partovi-Azar P., Petzold A., Lust E., Härk E. (2024). Empirical correlation of quantified hard carbon structural
parameters with electrochemical properties for sodium-ion batteries
using a combined WAXS and SANS analysis. Energy
Storage Mater..

[41] Jafta C. J., Petzold A., Risse S., Clemens D., Wallacher D., Goerigk G., Ballauff M. (2017). Correlating
pore size and shape to
local disorder in microporous carbon: A combined small angle neutron
and X-ray scattering study. Carbon.

[42] Härk E., Petzold A., Goerigk G., Risse S., Tallo I., Härmas R., Lust E., Ballauff M. (2019). Carbide derived carbons
investigated by small angle X-ray scattering: Inner surface and porosity
vs. graphitization. Carbon.

[43] Härk E., Petzold A., Goerigk G., Ballauff M., Kent B., Keiderling U., Palm R., Vaas I., Lust E. (2019). The effect
of a binder on porosity of the nanoporous RP-20 carbon. A combined
study by small angle X-ray and neutron scattering. Microporous Mesoporous Mater..

[44] Härk E., Ballauff M. (2020). Carbonaceous Materials
Investigated by Small-Angle
X-ray and Neutron Scattering. C.

[45] Zhang H., Hwang S., Wang M., Feng Z., Karakalos S., Luo L., Qiao Z., Xie X., Wang C., Su D. (2017). Single Atomic Iron Catalysts for Oxygen Reduction in Acidic Media:
Particle Size Control and Thermal Activation. J. Am. Chem. Soc..

[46] Wang X. X., Cullen D. A., Pan Y. T., Hwang S., Wang M., Feng Z., Wang J., Engelhard M. H., Zhang H., He Y. (2018). Nitrogen-Coordinated
Single Cobalt Atom Catalysts for Oxygen Reduction in Proton Exchange
Membrane Fuel Cells. Adv. Mater..

[47] Qu X., Han Y., Chen Y., Lin J., Li G., Yang J., Jiang Y., Sun S. (2021). Stepwise pyrolysis
treatment as an
efficient strategy to enhance the stability performance of Fe-NX/C
electrocatalyst towards oxygen reduction reaction and proton exchange
membrane fuel cell. Appl. Catal., B.

[48] Krumrey M., Ulm G. (2001). High-accuracy detector
calibration at the PTB four-crystal monochromator
beamline. NNucl. Instrum. Methods Phys. Res.,
Sect. A.

[49] Wernecke J., Gollwitzer C., Muller P., Krumrey M. (2014). Characterization
of
an in-vacuum PILATUS 1M detector. J. Synchrotron
Radiat..

[50] Blanton T. N., Huang T. C., Toraya H., Hubbard C. R., Robie S. B., Louër D., Göbel H. E., Will G., Gilles R., Raftery T. (1995). JCPDS—International
Centre for Diffraction Data
round robin study of silver behenate. A possible low-angle X-ray diffraction
calibration standard. Powder Diffr..

[51] Zhang F., Ilavsky J., Long G. G., Quintana J. P. G., Allen A. J., Jemian P. R. (2010). Glassy Carbon as
an Absolute Intensity Calibration
Standard for Small-Angle Scattering. Metall.
Mater. Trans. A.

[52] Keiderling U. (2002). The new ’BerSANS-PC’
software for reduction and treatment of small angle neutron scattering
data. Appl. Phys. A:Mater. Sci. Process..

[53] Stuhrmann H. B. (1985). Resonance
Scattering in Macromolecular Structure Research. Adv. Polym. Sci..

[54] Cromer D. T., Liberman D. A. (1981). Anomalous dispersion calculations
near to and on the
long-wavelength side of an absorption edge. Acta Crystallogr., Sect. A.

[55] Cromer D. T., Liberman D. (1970). Relativistic Calculation
of Anomalous Scattering Factors
for X Rays. J. Chem. Phys..

[56] Atomic Scattering Factors. https://henke.lbl.gov/optical_constants/asf.html.

[57] Fuchs D., Krumrey M., Müller P., Scholze F., Ulm G. (1995). High precision
soft x-ray reflectometer. Rev. Sci. Instrum..

[58] Sun Y. (2021). Anomalous
small-angle X-ray scattering for materials chemistry. Trends Chem..

[59] Taylor, G. W. ; Feigin, L. A. ; Svergun, D. I. Structure Analysis by Small-Angle X-Ray and Neutron Scattering; Springer Nature, 1987 10.1007/978-1-4757-6624-0.

