# Supplementary material for: Small-Angle X‑ray Scattering Monitoring of Porosity Evolution in Iron–Nitrogen–Carbon Electrocatalysts
Source: ACS Nano. 2025 Nov 14;19(46):40072–84. doi: 10.1021/acsnano.5c14955 (PMC12659431; doi:10.1021/acsnano.5c14955)
Supplement: Supplementary file 1 [file nn5c14955_si_001.pdf]

Supplementary Information

Small Angle X-ray Scattering Monitoring of Porosity Evolution in Iron–Nitrogen–Carbon Electrocatalysts

Rutha Jäger[1,a,*], Patrick Teppor[a], Armin Hoell[b], Uwe Keiderling[c], Christian Gollwitzer[d], Olga Volobujeva[e], Jaan Aruväli[f], Zdravko Kochovski[g], and Eneli Härk[1,g,*]

[a] Institute of Chemistry, Chair of Physical Chemistry, University of Tartu, Ravila 14A, 50411, Tartu, Estonia

[b] Department Structure and Dynamics of Energy Materials, Helmholtz-Zentrum Berlin für Materialien und Energie, Hahn-Meitner-Platz 1, 14109, Berlin, Germany

[c] Department Experiment Control and Data Acquisition, Helmholtz-Zentrum Berlin für Materialien und Energie, Hahn-Meitner-Platz 1, 14109, Berlin, Germany

[d] Physikalisch-Technische Bundesanstalt (PTB), Abbestr. 2-12, 10587 Berlin, Germany

[e] Department of Materials and Environmental Technology, Tallinn University of Technology, Ehitajate tee 5, 19086, Tallinn, Estonia

[f] Institute of Ecology and Earth Sciences, Department of Geology, University of Tartu, Ravila 14A, 50411, Tartu, Estonia

[g] Institute Electrochemical Energy Storage, Helmholtz-Zentrum Berlin für Materialien und Energie, Hahn-Meitner-Platz 1, 14109, Berlin, Germany

* Corresponding authors.

E-mail addresses: rutha.jager@ut.ee (R. Jäger), eneli.monerjan@helmholtz-berlin.de (E. Härk).

1 These authors contributed equally.

This file includes:

- Chemicals and materials used for Fe-N/Cat-X synthesis
- Methodology for evaluating electrochemical behavior
- Figure S1. Electrochemical characterization of electrocatalysts via RRDE
- Methodology - SEM, Low-temperature N_2 sorption, TEM, XRD, XPS
- Figure S2. Pore size distributions
- Figure S3. TEM images
- Figure S4. XRD patterns
- Table S1. XPS analysis data
- Figure S5. The theoretical values of the scattering factors calculated using the procedure described by Cromer and Libermann
- Structural model-free SAXS data analysis

- Figure S6. Separation of the fluctuation component and the ideal two-phase component of the system
- Table S2. Structural parameters derived from SAXS analysis
- References

Chemicals and materials used for Fe-N/Cat-X synthesis

Carbon source - powdered peat (Möllatsi Peatland, Estonia)
Nitrogen source - guanidine carbonate (99%, Sigma-Aldrich)
Iron salt - $Fe(NO_3)_3 \cdot 9H_2O$ (ACS reagent, ≥ 98%, Sigma-Aldrich)
Pore modifier - $ZnCl_2$ (anhydrous, ACS reagent, ≥ 97%, Sigma-Aldrich).
Hard template –hydroxyapatite (HA, $Ca_{10}(PO_4)_6(OH)_2$, ≥ 96%, 20 x 80 ± 10 nm particle size, Sigma-Aldrich)

Methodology for evaluating electrochemical behavior

Rotating ring-disk electrode (RRDE) measurements were performed to estimate the yield of hydrogen peroxide (x H_2O_2) formed in the ORR reaction. For this purpose, a catalyst suspension was prepared by ultrasonically homogenizing (for an hour) Fe-N/Cat-X catalyst with Milli-Q water, IPA, and Nafion® (D521 dispersion, Ion Power). The suspension was applied onto a glassy carbon disk (diameter 5 mm), achieving a catalyst loading of 0.3 mg cm^{-2}. In a three-electrode system, the working electrode was immersed in a 0.1 M KOH (pellets, 99.99%, Sigma-Aldrich) solution, using RHE and a graphite rod as reference and counter electrodes. The ORR activity was measured in the potential range from 1.2 to 0.3 V vs RHE (potential scan rate of 10 mV s^{-1}) using a Pine rotator and a Metrohm Autolab PGSTAT302 potentiostat equipped with a BA module and Nova 1.11 software. Before measuring the ORR curve, ring activation was performed by scanning the voltage from 1.4 to 0.025 V vs. RHE at a potential scan rate of 50 mV s^{-1} for 20 cycles, but the Pt ring was kept at a constant potential of 1.2 V vs. RHE when the ORR curves were measured. Electrochemical impedance spectroscopy was used to correct results for the iR-drop, and ORR polarization curves were recorded in O_2 and Ar-saturated solutions. The peroxide yield was calculated using the following equation: $x\,H_2O_2 = \frac{200 * \frac{I_{ring}}{I_{disk}}}{N + \frac{I_{ring}}{I_{disk}}}$, where I_{ring} and I_{disk} are the ring and disk currents, respectively, and N is the collection efficiency (0.249) determined for the electrode used in the experiment [1,2].

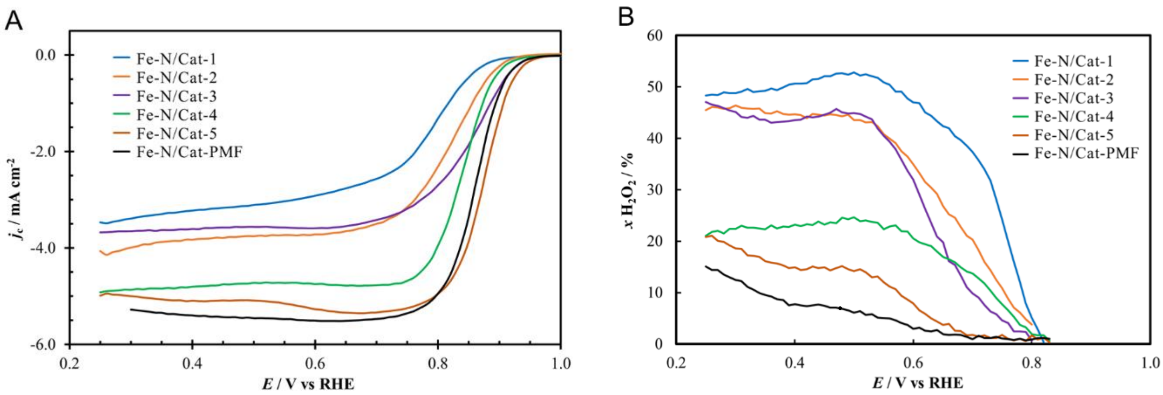

Figure S1. Electrochemical characterization of electrocatalysts via RRDE**.** a) ORR polarization curves in 0.1 M KOH solution at rotation rate of 1600 rpm (electrode potential scan rate of 10 mV s^{-1}). The catalyst loading was 0.3 mg cm^{-2}. b) Hydrogen peroxide yield measured for Fe-N/Cat-X catalysts.

Methodology – SEM, Low-temperature N_2 sorption, TEM, XRD, XPS

The scanning electron microscopy (SEM) analysis was conducted using a Zeiss Merlin microscope, SEM energy-dispersive X-ray spectroscopy (SEM-EDS) was performed using a Bruker EDX-XFlash® 6/30 detector. To prepare the samples, the Fe-N/Cat-X catalyst material was mixed with IPA, homogenized in an ultrasonic sound bath, and the sample was drop-casted onto copper substrates covered with carbon tape. The SEM measurements used an accelerating voltage of 4 kV, while SEM-EDS used 8 kV. Elemental compositions of the samples were determined using a P/B-ZAF standardless mode.

Low-temperature N_2 sorption measurements were carried out using Micromeritics ASAP 2020 and 3Flex instruments with nitrogen at its boiling point (-195.8 °C). Specific surface area (S_{DFT}) and degree of mesoporosity as indicated by the value (V_{micro}/V_{DFT}), where V_{micro} and V_{DFT} are micro pore volume and total pore volume, respectively, were obtained from pore size distribution data, which were computed using two-dimensional nonlocal density functional theory for heterogeneous surface (2D-NLDFT-HS) carbon materials via SAIEUS software.

Transmission electron microscopy (TEM) images for the Fe-N/Cat-X electrocatalysts were measured with a JEOL JEM-2100 device.

X-ray diffraction (XRD) measurements were performed using a Bruker D8 Advanced diffractometer with a Ni-filtered Cu Kα X-ray source. The resulting diffraction patterns were analyzed using Topaz software.

X-ray Photoelectron Spectroscopy (XPS) measurements were carried out at Turku University using a Thermo Scientific Nexsa system equipped with a monochromatized Al K-alpha radiation source (1486.6 eV). All N 1s photoelectron spectra were analyzed by curve fitting using the SPANCF package. [3,4]

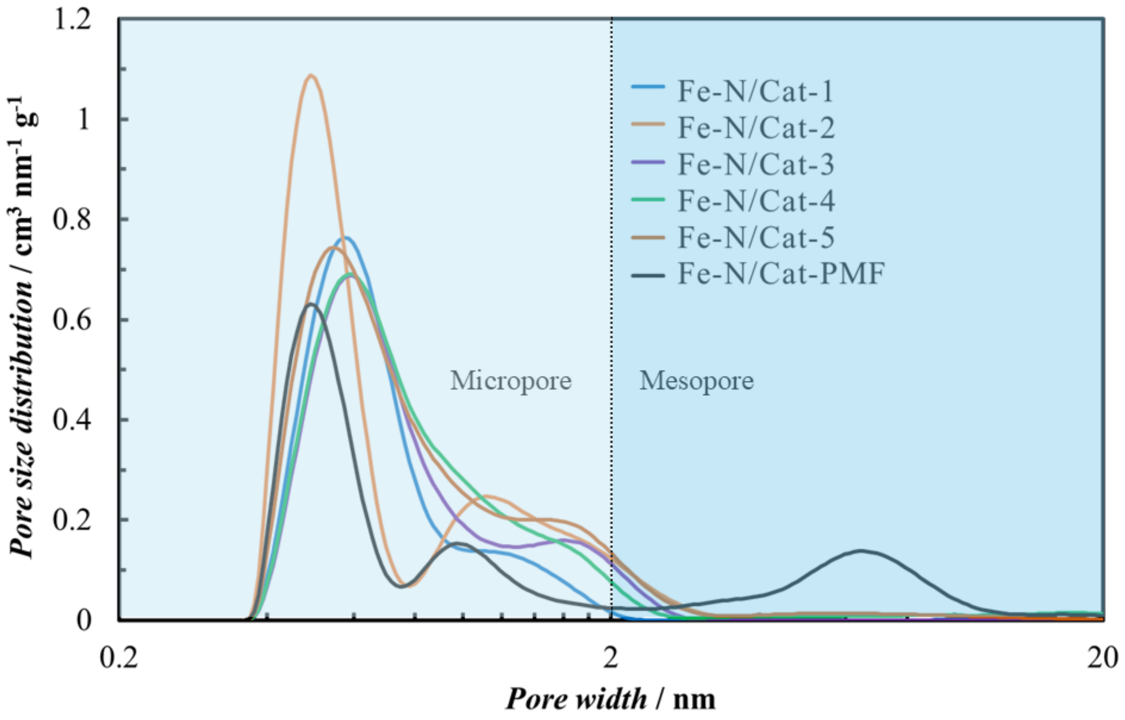

Figure S2. Pore size distributions obtained for the studied Fe-N/Cat-X electrocatalyst materials from N_2 sorption analysis.

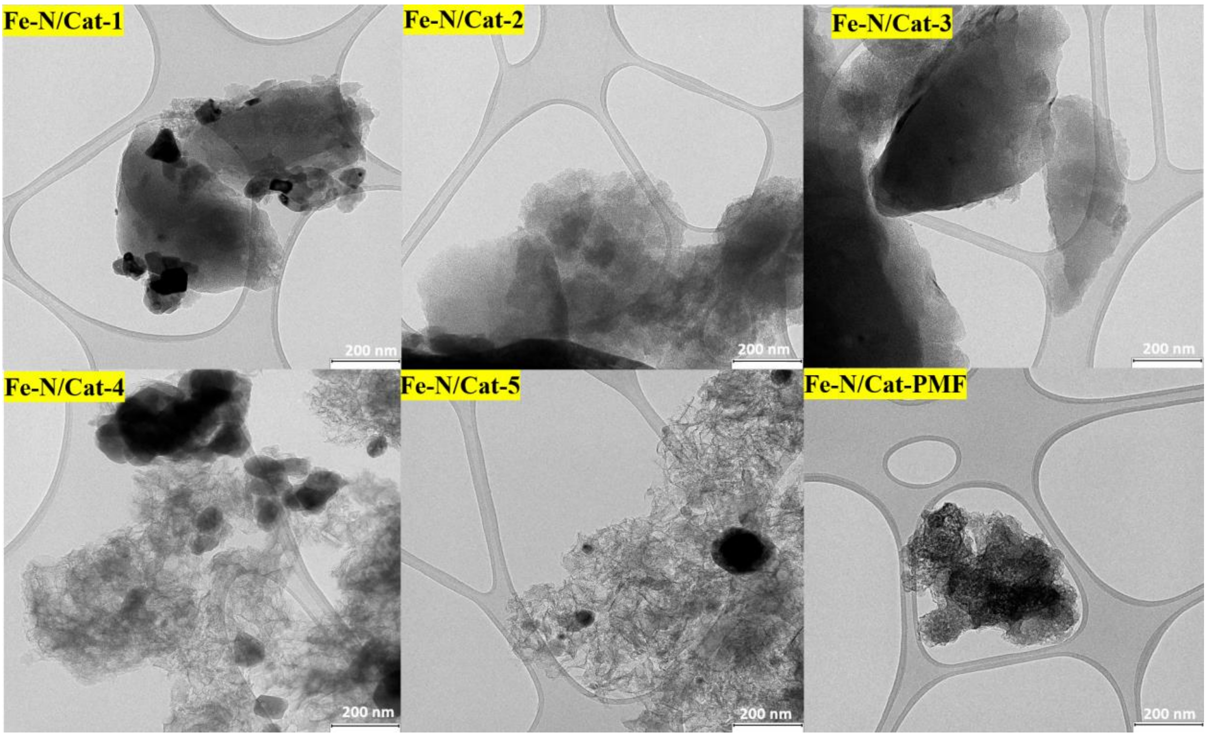

Figure S3. TEM images for Fe-N/Cat-X electrocatalysts at a magnification of 200 nm.

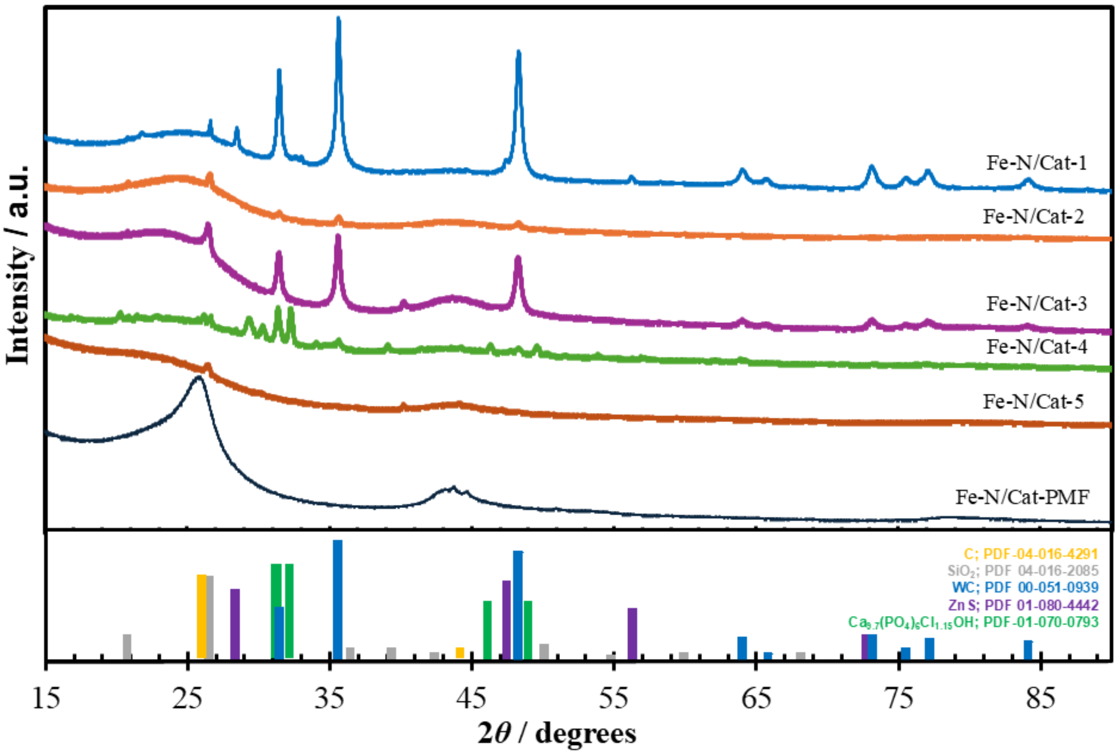

Figure S4. XRD patterns for Fe-N/Cat-X electrocatalysts.

Table S1. The total nitrogen content and the proportions of N form from fitting the N1s spectra measured with XPS.

	Total N (at%)	The proportions of N form				
		Pyridinic (%)	$Fe\text{-}N_x$ (%)	Pyrrolic (%)	Graphitic (%)	Pyridinic-oxide (%)
Fe-N/Cat-1	7.4	58	14	20	8	0
Fe-N/Cat-3	5.3	20	60	1	17	2
Fe-N/Cat-5	5.2	14	64	2	17	3
Fe-N/Cat-PMF	3.6	0	68	0	24	8

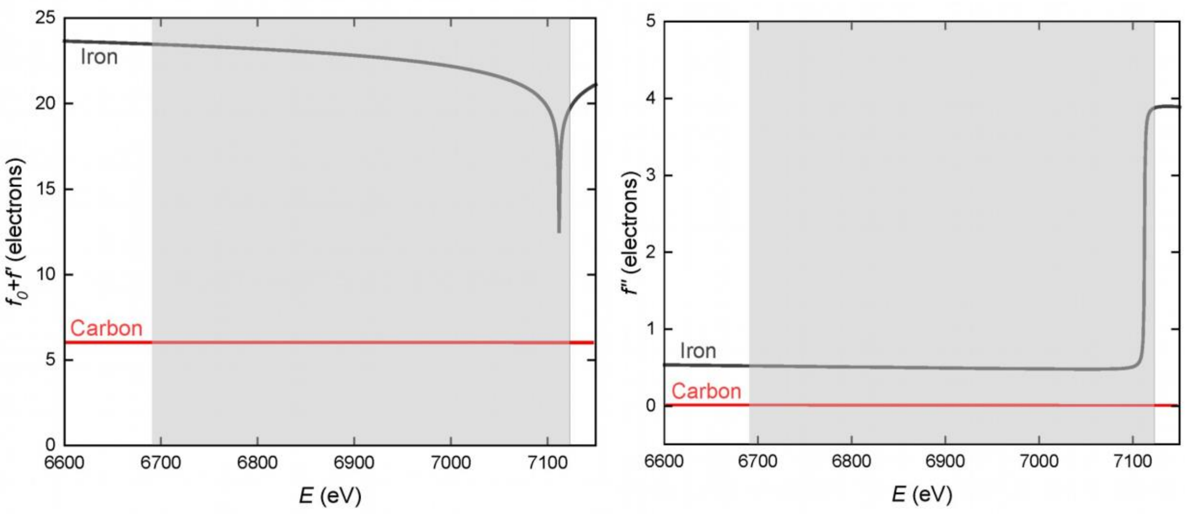

Figure S5. The theoretical values of the scattering factors calculated using the procedure described by Cromer and Libermann. a) Dependence of the real part (f_0+f') on the X-ray energy for iron and carbon elements with absorption edges in the range 6000 eV -7150 eV around the Fe K-edge. b) Dependence of the imaginary part (f'') of the X-ray scattering factor (f) on the X-ray energy for iron and carbon elements with absorption edges in the range 6000 eV-7150 eV around the Fe K-edge. In the ASAXS experiments, X-ray energies (6692, 6973, 7068, 7104, 7111, 7117, 7122, and 7124 eV) have been selected in the region indicated with the grey box.

Structural model-free SAXS data analysis

SAXS data curves were analyzed using a structural model-free analysis method combining Schiller, Mering, Perret, and Ruland approximations [5–7]. A detailed description of this approach can be found in the references [8-12].

The macroscopic differential scattering cross-section, $\frac{d\sigma}{d\Omega}(q)$, is normalised by mass:

$$\frac{d\Sigma_m}{d\Omega}(q) = \frac{1}{\rho_f}\frac{d\sigma}{d\Omega}(q), \text{(Equation S1)}$$

where ρ_f is the apparent filling density and $q = (4\pi\sin\theta/\lambda)$ is the modulus of the scattering vector with the wavelength λ and the scattering angle 2θ.

The mass normalized macroscopic scattering cross-section of porous carbon can be divided into three statistically independent scattering contributions [6,7,10,12]:

$$\frac{d\Sigma_m}{d\Omega}(q) = \frac{d\Sigma_{pores}}{d\Omega}(q) + \frac{d\Sigma_{fluct}}{d\Omega}(q) + C, \text{(Equation S2)}$$

where $\frac{d\Sigma_{pores}}{d\Omega}$ is the scattering of a two-phase system containing pores and a homogeneous carbon phase, and $\frac{d\Sigma_{fluct}}{d\Omega}$ is the scattering from the fluctuations in the carbon matrix caused by fluctuations in layer stacking and imperfections of the layers. The third component, C, is the contribution of the constant background that describes the q – independent 3D density fluctuations of an amorphous phase [10,11].

The fluctuation contribution can be approximated as [7,8,9,11]:

$$\frac{d\Sigma_{fluct}}{d\Omega}(q) = \frac{B_{fl} l_R^2 (18 + l_R^2 q^2)}{(9 + l_R^2 q^2)^2}, \text{(Equation S3)}$$

where B_{fl} defines the scattering contribution of the carbon phase, and l_R is a measure of the lateral dimensions of the graphene sheet, i.e., length of the graphene layer curvature.

Fluctuation term can be evaluated using modified Porod's law [6-8,13-15]. The intercept of the plot determines P_m and slope determines B_{fl} value [8,9]:

$$q^4 \frac{d\Sigma_m}{d\Omega}(q) = (2\pi)^4 P_m + B_{fl} q^2 + C q^4. \text{(Equation S4)}$$

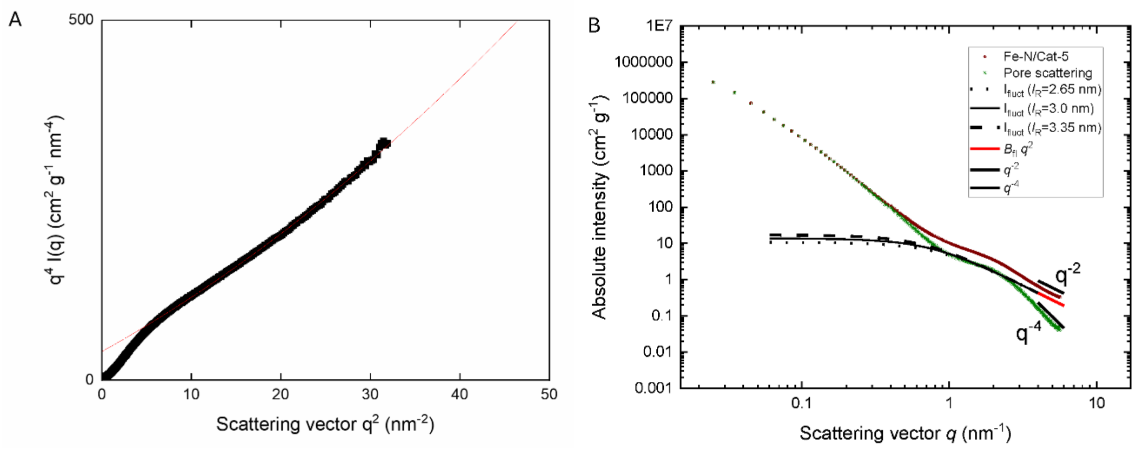

Figure S6. Separation of the fluctuation component and the ideal two-phase component of the system.

a) Analysis of the Modified Porod polynomial function of Fe-N/Cat-5 material as an example. The core of this analysis is the determination of the Porod constant, P_m, the fluctuation component, B_{fl}, the background component, C, and the Ruland length, l_R. P_m, B_{fl}, and C are obtained from a polynomial function, and the l_R component represents the point at which the experimental data begin to deviate from the polynomial function. b) The mass normalized

scattering intensities vs scattering vector q for Fe-N/Cat-5. The dashed lines show the limits of the fluctuation component for the l_R. The limits of the fluctuation component are set in such a way that the slope in the high q corresponds to q^{-4}, but that the fluctuation component does not exceed the macroscopic scattering cross-section. l_R is the average of the limit values (noted in the figure) and the limits are taken into account as the absolute deviation of the l_R.

The invariant Q_m sum for the pore scattering [7] is related to the porosity ϕ:

$$Q_m = \frac{\Delta\rho^2}{\rho_{grain}}\phi(1-\phi), \text{(Equation S5)}$$

where ρ_{grain} is the overall mass density of the two-phase system that is connected to the skeletal density, ρ_{sk}, via $\rho_{grain} = \rho_{sk}(1-\phi)$ and its value is higher than the apparent filling density, ρ_f, because it does not include empty spaces between grains [8-12]. Scattering contrast, $\Delta\rho$ is calculated from skeletal density.

Porod length l_p was calculated as:

$$l_p = \frac{Q_m}{2\pi^3 P_m}. \text{(Equation S6)}$$

This analysis leads directly to the inner surface S/m:

$$P_m = \frac{\Delta\rho^2}{2\pi^3}\frac{S}{m}, \text{(Equation S7)}$$

where $\Delta\rho$ denotes the scattering contrast between the vacuum and the electrocatalyst.

The mean chord length of the pores, l_{pore}, and the mean chord length of the carbon matrix, l_{solid}, were calculated as:

$$l_{pore} = \frac{l_p}{1-\phi};\ l_{solid} = \frac{l_p}{\phi}. \text{(Equation S8)}$$

The weight averaged chord length, l_c, was calculated via:

$$l_c = \frac{1}{2\pi Q_m}\int_0^\infty \frac{d\Sigma_m}{d\Omega}(q)q\,dq. \text{(Equation S9)}$$

The degree of disorder (DoD) was calculated by combining lateral imperfections and the invariant Q_m as following [5]:

$$\frac{B_{fl}\phi}{2\pi a_3 Q_m} = \frac{\langle\Delta^2 a_3\rangle}{\langle a_3\rangle^2} + \frac{\langle\Delta^2 l_R\rangle}{\langle l_R\rangle^2}, \text{(Equation S10)}$$

where a_3 is the distance between graphene layers [16,17].

Table S2. Extended structural parameters derived from SAXS analysis.

Parameter	Fe-N/Cat-1	Fe-N/Cat-2	Fe-N/Cat-3	Fe-N/Cat-4	Fe-N/Cat-5	Fe-N/Cat-PMF
C (cm^2g^{-1})	0.051 ± 0.002	0.038 ± 0.003	0.04 ± 0.004	0.096 ± 0.002	0.646 ± 0.003	0.062 ± 0.002
B_{fl} ($cm^2nm^{-2}g^{-1}$)	1.34 ± 0.11	3.32 ± 0.09	3.51 ± 0.16	4.23 ± 0.07	6.89 ± 0.12	2.18 ± 0.08
P_m ($cm^2g^{-1}nm^{-4}$)	0.004 ± 0.0007	0.023 ± 0.0006	0.02 ± 0.001	0.009 ± 0.0004	0.025 ± 0.0006	0.02 ± 0.0004
S/m (m^2 g^{-1})	36.7 ± 7.3	193.0 ± 22.2	196.0 ± 22.6	100.9 ± 13.1	214.7 ± 23.9	168.5 ± 18.5
l_R (nm)	1.7	1.4	2.3	1.3	3.0	7.0
Q_m ($cm^2g^{-1}nm^{-3}$)	0.45 ± 0.11	1.54 ± 0.10	1.54 ± 0.13	2.01 ± 0.13	2.74 ± 0.14	2.78 ± 0.11
ϕ	0.03 ± 0.007	0.10 ± 0.009	0.10 ± 0.01	0.14 ± 0.001	0.18 ± 0.001	0.19 ± 0.001
l_p (nm)	1.741 ± 0.505	1.095 ± 0.078	1.032 ± 0.100	3.477 ± 0.263	1.735 ± 0.097	2.198 ± 0.100
l_{pore} (nm)	1.79 ± 1.07	1.22 ± 0.14	1.15 ± 0.170	4.04 ± 1.27	2.13 ± 0.28	2.71 ± 0.35
l_{solid} (nm)	59.2 ± 22.4	10.50 ± 1.16	10.04 ± 1.40	24.86 ± 2.83	9.4 ± 0.9	11.71 ± 0.95
l_c (nm)	16.7 ± 4.02	4.25 ± 0.32	8.06 ± 0.73	25.53 ± 1.73	17.6 ± 0.94	12.37 ± 0.53
l_c/l_p	9.62 ± 3.62	3.88 ± 0.4	7.81 ± 1.04	7.34 ± 0.75	10.2 ± 0.8	5.63 ± 0.35
$\frac{\Delta^2 a_3}{a_3^2}+\frac{\Delta^2 l_R}{l_R^2}$	0.04 ± 0.015	0.107 ± 0.012	0.111 ± 0.016	0.14 ± 0.015	0.22 ± 0.02	0.07 ± 0.006

C – background scattering; B_{fl} – fluctuation component; P_m – Porod constant; S/m – internal surface area per mass; l_R – length of the graphene layer curvature; Q_m – invariant Q; ϕ – porosity; l_p – Porod length; l_{pore} – average chord length of pores; l_{solid} – average chord length of pore walls; l_c – average chord length; l_c/l_p – anisometric ratio; $\frac{\Delta^2 a_3}{a_3^2}+\frac{\Delta^2 l_R}{l_R^2}$ – degree of disorder (DoD).

References

[1] A. Bonakdarpour, M. Lefevre, R. Yang, F. Jaouen, T. Dahn, J.-P. Dodelet and J.R. Dahn, Impact of loading in RRDE experiments on Fe-N-C catalysts: Two or four electron oxygen reduction?, Electrochemical and Solid State Letters, 11 (2008) B105-B108. DOI 10.1149/1.2904768

[2] L. Bouleau, S. Pérez-Rodríguez, J. Quílez-Bermejo, M.T. Izquierdo, F. Xu, V. Fierro, A. Celzard, Best practices for ORR performance evaluation of metal-free porous carbon electrocatalysts, Carbon. 189 (2022) 349–361. https://doi.org/10.1016/j.carbon.2021.12.078.

[3] E. Kukk, K. Ueda, U. Hergenhahn, X.J. Liu, G. Prümper, H. Yoshida, Y. Tamenori, C.-Makochekanwa, T. Tanaka, M. Kitajima, H. Tanaka, Violation of the Franck-Condon principle due to recoil effects in high energy molecular core-level photoionization. Phys Rev Lett. (2005) Sep 23;95(13):133001. doi: 10.1103/PhysRevLett.95.133001.

[4] E. Kukk, G. Snell, J.D. Bozek, W.-T. Cheng, N. Berrah, Vibrational structure and partial rates of resonant Auger decay of the core excitations in nitric oxide, Phys. Rev. A. 63 (2001) 062702. https://doi.org/10.1103/PhysRevA.63.062702.

[5] W. Ruland, Small-angle scattering of two-phase systems: determination and significance of systematic deviations from Porod's law, J. Appl. Crystallogr. 4 (1971) 70–73. https://doi.org/10.1107/S0021889871006265.

[6] C. Schiller, J. Mering, P. Cornuault, F. Du Chaffaut, Defauts structuraux dans les carbones graphitables effets des traitements thermiques—partie II, Carbon. 5 (1967) 507–516. https://doi.org/10.1016/0008-6223(67)90027-9.

[7] R. Perret, W. Ruland, X-ray small-angle scattering of non-graphitizable carbons, J. Appl. Crystallogr. 1 (1968) 308–313. https://doi.org/10.1107/S0021889868005558.

[8] C.J. Jafta, A. Petzold, S. Risse, D. Clemens, D. Wallacher, G. Goerigk, M. Ballauff, Correlating pore size and shape to local disorder in microporous carbon: A combined small angle neutron and X-ray scattering study, Carbon. 123 (2017) 440–447. https://doi.org/10.1016/j.carbon.2017.07.046.

[9] E. Härk, A. Petzold, G. Goerigk, S. Risse, I. Tallo, R. Härmas, E. Lust, M. Ballauff, Carbide derived carbons investigated by small angle X-ray scattering: Inner surface and porosity vs. graphitization, Carbon. 146 (2019) 284–292. https://doi.org/10.1016/j.carbon.2019.01.076.

[10] E. Härk, A. Petzold, G. Goerigk, M. Ballauff, B. Kent, U. Keiderling, R. Palm, I. Vaas, E. Lust, The effect of a binder on porosity of the nanoporous RP-20 carbon. A combined study by small angle X-ray and neutron scattering, Microporous Mesoporous Mater. 275 (2019) 139–146. https://doi.org/10.1016/j.micromeso.2018.08.022.

[11] E. Härk, M. Ballauff, Carbonaceous Materials Investigated by Small-Angle X-ray and Neutron Scattering, C. 6 (2020) 82. https://doi.org/10.3390/c6040082.

[12] Kalder, L.; Olgo,A.; Lührs,J.; Härmas, R.; Aruväli, J.; Partovi-Azar,P.; Petzold,A.; Lust,E.; Härk,E. (2024). Empirical correlation of quantified hard carbon structural parameters with electrochemical properties for sodium-ion batteries using a combined WAXS and SANS analysis, Energy Storage Materials, 67,103272, DOI:10.1016/j.ensm.2024.103272.

[13] B. Smarsly, M. Antonietti, T. Wolff, Evaluation of the small-angle x-ray scattering of carbons using parametrization methods, J. Chem. Phys. 116 (2002) 2618–2627. https://doi.org/10.1063/1.1433463.

[14] R. Perret, W. Ruland, X-ray small-angle scattering of glassy carbon, J. Appl. Crystallogr. 5 (1972) 183–187. https://doi.org/10.1107/S0021889872009161.

[15] O. Glatter, O. Kratky, H.C. Kratky, Small angle X-ray scattering, Academic Press, 1982.

[16] R.E. Franklin, J.T. Randall, Crystallite growth in graphitizing and non-graphitizing carbons, Proc. R. Soc. Lond. Ser. Math. Phys. Sci. 209 (1951) 196–218. https://doi.org/10.1098/rspa.1951.0197.

[17] R.E. Franklin, The structure of graphitic carbons, Acta Crystallogr. 4 (1951) 253–261. https://doi.org/10.1107/S0365110X51000842.
